# Schistosome species, parasite development, and co-infection combinations determine microbiome dynamics in the snail *Biomphalaria glabrata*

**DOI:** 10.1186/s42523-025-00471-3

**Published:** 2025-10-02

**Authors:** Ruben Schols, Cyril Hammoud, Karen Bisschop, Isabel Vanoverberghe, Tine Huyse, Ellen Decaestecker

**Affiliations:** 1 Department of Biology, Royal Museum for Central Africa, Tervuren, 3080 Belgium; 2 Laboratory of Aquatic Biology, Microbiome EcoEvo Unit, KU Leuven, Campus Kortrijk, Kortrijk, 8500 Belgium; 3 Limnology Unit, Department of Biology, Ghent University, Ghent, 9000 Belgium; 4 Royal Netherlands Institute for Sea Research, Den Hoorn, 1797 SZ The Netherlands; 5 Terrestrial Ecology, Department of Biology, Ghent University, Ghent, Belgium; 6 Division of Biodiversity and Evolution, Department of Biology, Lund University, Lund, Sweden

## Abstract

**Background:**

Schistosomiasis is a snail-borne disease affecting over 200 million people worldwide. Despite dedicated control efforts and effective diagnostic tools, schistosomiasis remains prevalent. Novel and sustainable control measures are urgently needed. Bacteria might offer such a solution as links between bacteria, disease resistance and transmission potential of intermediate hosts have been established in other systems. To better understand the tripartite interaction potentially driving snail-schistosome compatibility patterns, microbial communities must be investigated throughout and across various parasite exposure conditions. Therefore, we studied *Biomphalaria glabrata* snails exposed to a high- and low-shedder population of *Schistosoma mansoni* and *Schistosoma rodhaini* in single and co-exposure experiments. Snails were sacrificed at different time points post-exposure and their bacterial communities and trematode (co-)infection status were determined through metabarcoding tools.

**Results:**

Snails infected by low- and high-shedder *S. mansoni* populations were more likely to have bacterial community dysbiosis than those infected by *S. rodhaini* but this was also affected by miracidial load. Moreover, the single-infection hierarchical effect on the bacterial component of the microbiome is not maintained under co-infection with *S. rodhaini*, which appears to stabilize the snail’s bacterial profile even after being outcompeted by high-shedder *S. mansoni*. Finally, alpha diversity differed significantly between infected and uninfected snails around the onset period of shedding at 30 days post-miracidial exposure.

**Conclusion:**

The timing of this bacterial shift suggests an intricate parasite-snail interaction around key parasite development moments. Future studies investigating the tripartite interaction are advised to consider the effect of outcompeted or prepatent infections on the snail’s microbiome.

**Supplementary Information:**

The online version contains supplementary material available at 10.1186/s42523-025-00471-3.

## Background

Freshwater snails play a central role in the transmission of parasitic diseases, serving as intermediate hosts for trematodes that affect both human and animal populations [1]. Among these snail-borne diseases, schistosomiasis - a neglected tropical disease caused by blood flukes of the genus *Schistosoma* - carries the greatest global health burden, affecting over 200 million people globally [2]. Schistosomiasis manifests in two main clinical forms: urogenital schistosomiasis, caused predominantly by *Schistosoma haematobium*; and intestinal schistosomiasis, caused by species such as *Schistosoma mansoni* and *Schistosoma japonicum* [3–5]. The intestinal form, responsible for symptoms such as bloody stool, diarrhea, and anemia, is most commonly associated with *S. mansoni*, which relies on freshwater snails of the genus *Biomphalaria* as intermediate host (Suppl. Figure 1 [3, 4]), . However, not all snail strains transmit schistosomes equally well, providing an interesting landscape to better understand compatibility patterns, as currently the driving factors often remain ill-understood [6, 7]. Further confounding the compatibility between schistosomes and their snail hosts are co-infections with different populations of the same species, different schistosome species and other co-infecting trematode species in a single snail specimen (e.g [8–12]). Such co-infections could affect disease outcome through immune priming of the snail host [13] or synergistic or antagonistic parasite-parasite interactions [10–12, 14]. At the intraspecific level, co-infections by up to nine genotypes of *S. mansoni* are possible, leading to reduced individual fitness due to reproductive competition [9, 15]. At the interspecific level of schistosome species, the rodent infecting *Schistosoma rodhaini* has a partially overlapping distribution and host range with *S. mansoni* potentially influencing the transmission of both schistosomes [16]. Nevertheless, *S. mansoni* and *S. rodhaini* co-infections in *Biomphalaria pfeifferi* and *Biomphalaria sudanica* did not show an altered cercarial emergence compared to single infections [16]. In contrast, co-infection experiments in *Biomphalaria glabrata* by both schistosome species did reveal a shift in the timing of cercarial emergence and a reduction in the number of released cercariae compared to single infection with and without patent co-infection [12]. At the interspecific level of trematode species, an interesting two-way interaction was reported by Laidemitt and colleagues [11] whereby the cattle amphistome *Calicophoron sukari* requires an earlier infection of *S. mansoni* in *B. pfeifferi* to establish itself but will in turn inhibit further development of *S. mansoni.* Moreover, such interactions illustrate that the control of one disease could inadvertently lead to the increase of another [11]. Therefore, including parasite-parasite interactions within snail-parasite compatibility studies becomes imperative.

A host and its microbiome are intimately linked in what is known as the ‘holobiont’ [17], of which the microbial component constitutes a rapidly evolving facet [18, 19]. Various factors, such as environment, food, disease and age can influence the microbiome [20–22]. Such microbiome alterations can help the acclimatization or adaptation of a host but can also worsen the effect an environmental stressor has on a host in what is known as ‘dysbiosis’ [22]. Interestingly, the dysbiotic state is frequently linked to disease [22–24] and may cascade down to the immune system facilitating pathogen colonization and growth [23]. We define the dysbiotic state as an increased stochasticity in the microbial community of stressed individuals compared to healthy individuals, which is measured by an increased within-treatment Beta diversity [25].

The microbiome has previously been linked to disease resistance and thereby the transmission potential of hosts in the host-pathogen-microbiome tripartite interaction [26, 27]. Within the snail-trematode-microbiome interaction framework, Chernin [28] already explored the effect of bacteria on infection outcome, revealing that *B. glabrata* snails lacking viable bacteria and control snails were equally susceptible to *S. mansoni* [28]. Nevertheless, the current knowledge on the microbiome of intermediate snail hosts of schistosomes remains limited (see review of Sun et al. [29]).

To establish an infection, a miracidium (the larval stage released from schistosome eggs that is infective to snails) penetrates the head-foot region of *Biomphalaria* snails and rapidly transforms to the next developmental stage, a sporocyst [30]. During this stage, the parasite has to escape the internal defence system of the snail by modulating hemocyte activity [31, 32]. Hemocytes of resistant snails encapsulate the recently transformed sporocyst within their tissue and attempt to kill the parasite [31]. Such a response is not adequate in susceptible snails and in the next two to three weeks mother sporocysts generate clonal daughter sporocysts, still located in the head-foot region [33]. Next, daughter sporocysts migrate to the gonad-digestive gland region to further multiply asexually, inflicting significant damage in the process [34, 35]. This damage leads to partial or complete destruction of ovotestis tissue, typically castrating the snail host [34, 35]. Cercariae (the larval stage infective to final hosts) require arginine and glucose to mature within the daughter sporocyst thereby continuously absorbing large energy reserve of the gonad-digestive gland region during their development [35]. Combined, these processes reveal a direct (destruction of tissue) and indirect (snail starvation through nutrient absorption) effect on snail fitness [35]. However, once matured, cercariae require only a minimal supply of energy for migration and emergence which happens approximately four weeks after miracidial exposure of the snail [35].

**Fig. 1:**
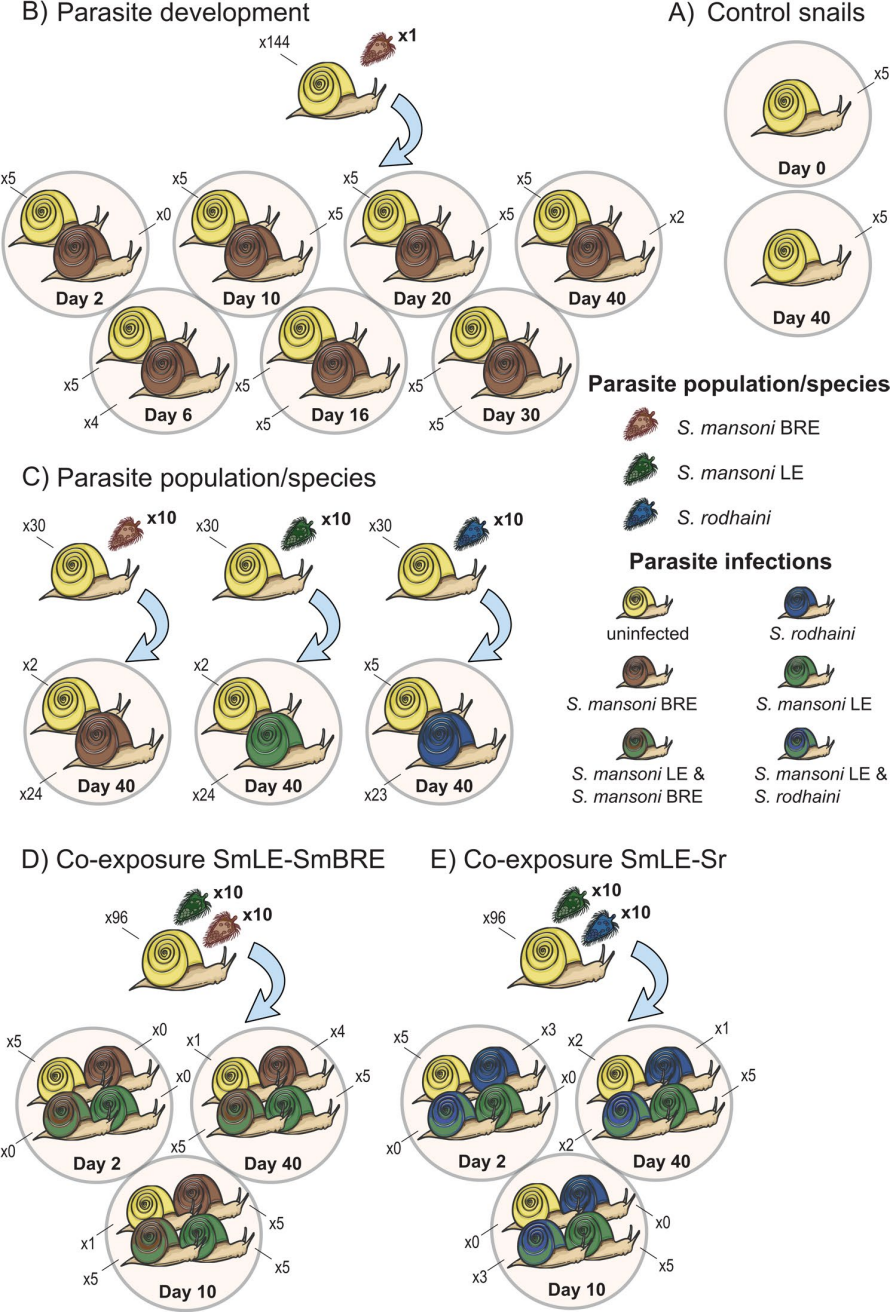
Experiment setup. Miracidia of *Schistosoma mansoni* population Recife (SmBre, low shedder, brown), Belo Horizonte (SmLE, high shedder, green), and *Schistosoma rodhaini* (blue) were obtained from experimentally infected mice. (**A**) control snails were collected from the donor population at day 0 and day 40 of the experiment. (**B**) *Biomphalaria glabrata* snails were exposed to single miracidia of *S. mansoni* population Bre and sacrificed 2, 6, 10, 16, 20, 30 and 40 days post-miracidia exposure. (**C**) *B. glabrata* snails were exposed to 10 miracidia of either *S. mansoni* Bre, *S. mansoni* LE or *S. rodhaini* and sacrificed 40 days post-miracidia exposure. (**D**) *B. glabrata* snails were co-exposed to 10 miracidia of *S. mansoni* LE and *S. mansoni* BRE and sacrificed 2, 10 and 40 days post-miracidia exposure. (**E**) *B. glabrata* snails were co-exposed to 10 miracidia of *S. mansoni* LE and *S. rodhaini* and sacrificed 2, 10 and 40 days post-miracidia exposure. Control snails were never exposed to miracidia and collected at day 0 and 40. Color codes of snail shells correspond to the infection status as determined by the HTAS protocol of Hammoud et al. [36]. The numbers on top (144 in B, 30*3 in C, 96 in D, and 96 in E) indicate the initial sample sizes, while the numbers showing the treatments are the final samples of which the bacterial component of the microbiome was characterized through 16S rRNA gene metabarcoding in this study

**Fig. 2:**
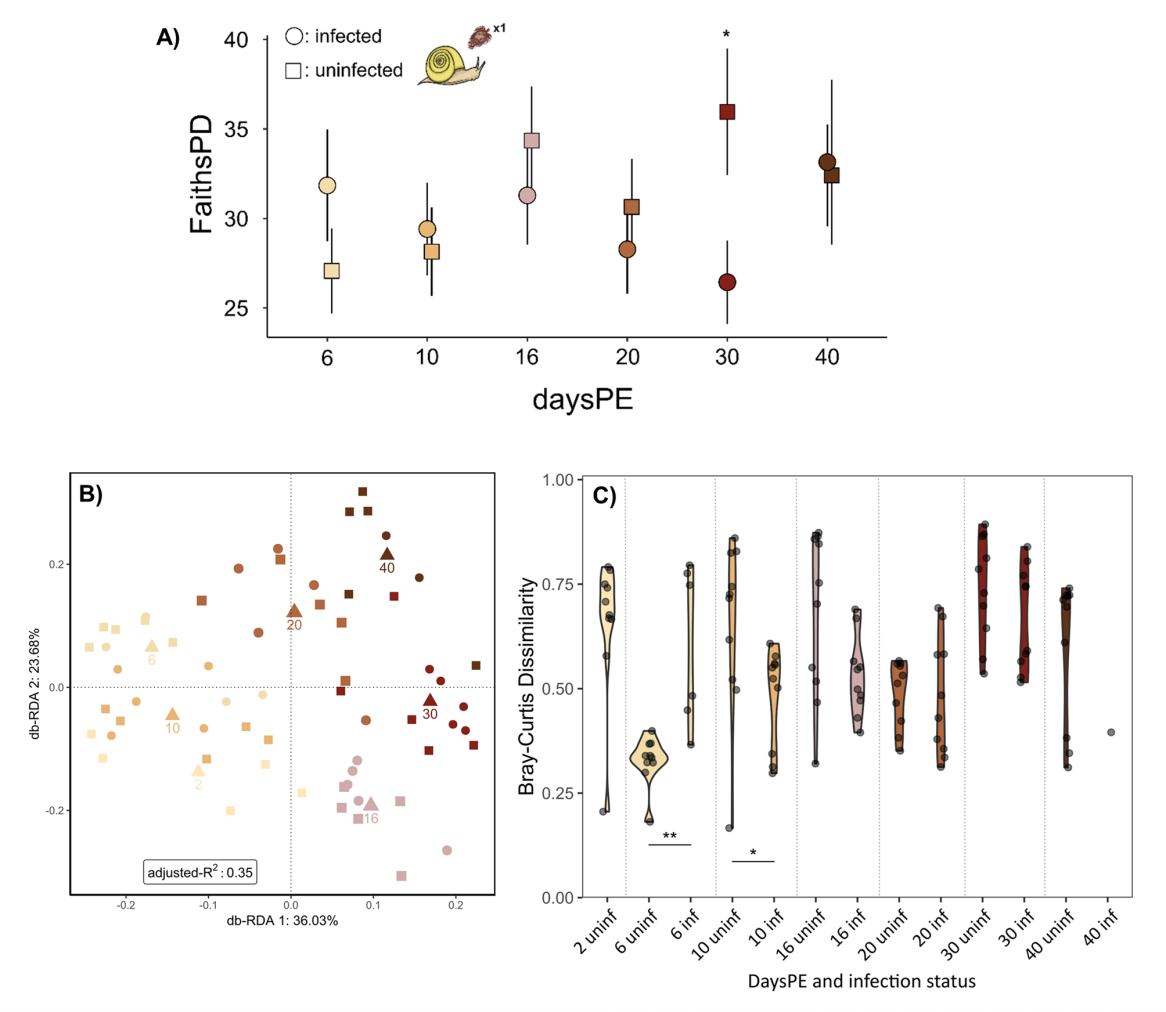
The alpha and beta diversity of infected vs uninfected samples across various days post SmBRE miracidium exposure (daysPE). **A**) Faith’s phylogenetic diversity metric (FaithsPD), based on mean values with error bars representing a single standard deviation. **B**) Bray-Curtis dissimilarity measure as shown by RDA plot of the bacterial community adjusted for the different days post exposure. Infection status was selected out of the RDA by ordistep but is indicated for illustration purposes. The triangles are the centroids of all samples belonging to the specific post exposure day. **C**) Violin plots showing the within-group Bray–Curtis dissimilarity with labels on the x-axis indicating the days post exposure (first number) followed by infection status (uninf = uninfected, inf = infected). All samples at 2 daysPE are classified as uninfected since the infection status cannot be reliably determined this soon after exposure. Circles indicate infected samples, squares uninfected samples. Significance: <0.05=‘*’ and < 0.01=‘**’. Sample counts per condition can be found in Fig. 1 and Suppl. Table 1

**Fig. 3:**
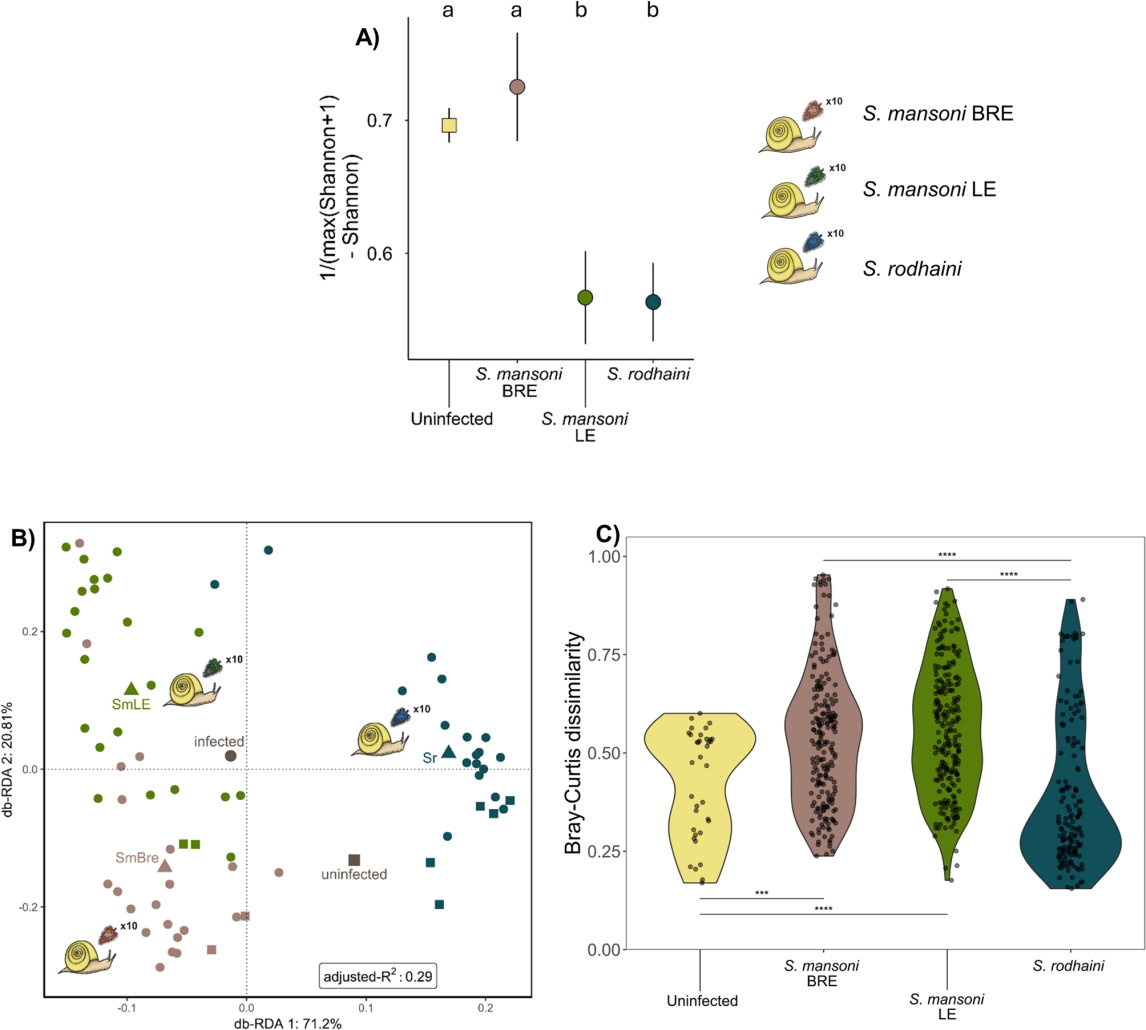
The bacterial aspect of the microbiome for snails 40 days post exposure to *S. mansoni* (SmBRE, low shedder), *S. mansoni* (SmLE, high shedder), and *S. rodhaini* (Sr). Uninfected samples from all exposure experiments are combined under “Uninfected” as selected by the model. **A**) Inverse transformed Shannon diversity metric (alpha diversity) for snails 40 days post exposure to SmBRE (*S. mansoni* low shedder), SmLE (*S. mansoni* high shedder), and Sr (*S. rodhaini*). Significant differences are indicated by letter groups. **B**) Bray-Curtis dissimilarity measure of the bacterial community across the different parasite exposures as shown by RDA plot. The RDA considers parasite exposure and infection status additively. Ordistep excluded the interaction term. The triangles indicate centroid values for each parasite exposure, the large grey circle the centroid for all infected samples, and the large grey square the centroid for all uninfected samples. Circles indicate infected samples, squares uninfected samples. **C**) The within-group Bray–Curtis dissimilarity for the different diagnosed infections. Significance: <0.05=‘*’, < 0.01=‘**’, < 0.0001=‘****’. Sample counts per condition can be found in Fig. 1 and Suppl. Table 1

The snail samples utilized for bacterial characterization in our study were sourced from the research conducted by Hammoud and colleagues [36]. Their research involved exposure experiments of *B. glabrata* to three schistosome populations - two *S. mansoni* and one *S. rodhaini* - for which (co-)infection status is known via diagnostic PCRs and metabarcoding [36, 37]. This created an opportunity to delve deeper into the microbial aspects of the experiment. By characterizing the bacterial component of the microbiome we aim to (1) compare bacterial community composition between infected and uninfected snails, and explore the composition throughout parasite development (Fig. 1B), (2) evaluate the impact of single infections by different parasite populations and species on bacterial profiles (Fig. 1C), and (3) explore the effects of co-infections and their development on the microbiome (Fig. 1D & E). These insights will provide the necessary foundation for targeted transplant experiments and contribute to the limited knowledge on the tripartite interaction between snails, parasites and the microbiome. We hypothesized that (1) infected and uninfected snails differ in microbiome composition and diversity, resulting either from microbiome-mediated variation in host resistance or from infection-induced shifts in snail immune responses, with or without parasite mediation [31, 38, 39]; (2) key parasite development stages correspond to significant shifts in the microbiome due to mechanical damage and broader physiological and immunological changes to the snail [31, 32, 34, 35]; (3) *S. mansoni* population LE exerts a stronger impact on the snail microbiome due to its greater disruption of host physiology compared to *S. mansoni* population BRE [40]; and (4) *S. mansoni* population LE dominates microbiome signatures regardless of co-infection with *S. mansoni* population BRE or *S. rodhaini*, due to its competitive advantage over both populations [12, 36].

## Materials and methods

### Experimental conditions

Snail samples used for bacterial characterization were obtained from the study of Hammoud and colleagues [36] which was conducted in early 2018 and therefore followed the same experimental setup (Fig. 1, Suppl. Tables 1 and Suppl. Table 2). Briefly, laboratory-reared snails of *B. glabrata* population BgGUA2 of the same age cohort (5–7 mm) from Guadeloupe, Dans Fond, were simultaneously exposed to various allopatric combinations of *S. mansoni* population BRE (Brazil, Recife, low-shedder, from here on referred to as SmBRE), *S. mansoni* population LE (Brazil, Belo Horizonte, high-shedder, from here on referred to as SmLE), and *S. rodhaini* (Burundi, from here on referred to as Sr) at the Host-Pathogen-Environment Interactions laboratory of the University of Perpignan (UPVD, France). BgGUA2 is a highly permissive snail population and the selected parasite populations (SmLE, SmBRE, Sr) are highly infective [36, 41, 42], ensuring a sufficiently high infection rate for comparative purposes. The snail and parasite populations have been in laboratory cycling for several decades [36, 41, 42]. Parasite populations have been maintained through Swiss OFI mice as final hosts and their sympatric snail intermediate host: SmBRE with *B. glabrata* population BRE, SmLE with *B. glabrata* population Barreiro and Sr with *Biomphalaria pfeifferi*. Miracidia were obtained from schistosome infections in the mouse strain Swiss OFI whereby eggs were isolated from the liver and allowed to hatch in water. Miracidia were allowed to infect individual snails for 24 h. To address the three abovementioned objectives, four different sub-experiments were conducted. A first sub-experiment was performed to characterize the detectability of parasites during infection development, involving 144 snails each individually exposed to a single SmBRE miracidium where specimens were sacrificed 2, 6, 10, 16, 20, 30 and 40 days post-parasite exposure (Fig. 1B; time points correspond to key developmental stages: detection of aborted infections [day 2; [36]], onset of proliferation [day 6; [43]], immune modulation and tissue reorganization [day 10; [43]], daughter sporocysts migration [day 16; [33, 44]], tissue remodeling and hemocyte exclusion zones [day 20; [33]], onset of shedding [day 30; [36, 40, 45–47]], and post-peak cercarial release [day 40; [40, 47]]). The gradual development of SmBRE facilitates tracking of the progressive build-up of infection over time [36, 40]. Another sub-experiment focused on the detectability of various parasite species, involving 30 snails individually exposed to 10 miracidia of either of the three schistosome populations and sample collection at 40 days post-parasite exposure (Fig. 1C). Finally, two last sub-experiments aimed at characterizing co-infections by SmLE and SmBRE, and SmLE and Sr, involving 96 snails individually exposed to 10 miracidia of each parasite population and samples being collected at 2, 10 and 40 days post-parasite exposure (Fig. 1D & E). The timepoints were selected to represent a view on the microbiome during infection establishment (day 2), during co-infection (day 10), and after competitive exclusion (day 40). Control snail specimens of the same age cohort were collected from the donor population on day 0 (*n* = 5) and day 40 (*n* = 5) of the experiment. Across sub-experiments, snails were maintained in a single aquarium per exposure experiment - allowing identical environmental conditions for infected and uninfected snails per treatment - in 10 L aquaria at 25 °C under a 12 h:12 h light-dark regime with constant aeration. Snails were fed *ad libitum* with washed pesticide-free salad and water was replaced once a week with borehole water. Snails were killed by heat shock (70 °C for 1 min) and stored in 80% ethanol.

Next, the whole soft tissue of the snail was isolated from the shell by pulling on the foot using a sterile needle. DNA was then extracted from the soft tissue (i.e. without shell) with the E.Z.N.A. ^®^Mollusc DNA Kit (OMEGA Bio-tek, Norcross, GA, USA) according to the manufacturer’s guidelines and stored at -21 °C. The infection status (infected yes or no) was determined through the adapted infection RD-PCR of Schols and colleagues [37] whereby the PCR cycles were reduced to 25 to avoid the detection of aborted infections as determined by Hammoud and colleagues [36]. (Co)infections were then characterized through the metabarcoding approach [36].

### 16S rRNA gene metabarcoding

A total of 212 samples (See Suppl. Table 1), four bacterial mock communities, one extraction negative and two PCR negatives were included in the 16S rRNA sequencing in a protocol adapted from Schols and colleagues [48]. Briefly, the pipeline targets the V3-V4 regions and is adapted from [49] and [50]. PCR reactions were done in triplicate in 25 µl: 12.5 µl of PCRBIO HS VeriFi Mix (PCR Biosystems), 0.75 µl of primer 341F (5’-CCTACGGGNGGCWGCAG-3’; [51]) and 0.75 µl of primer 805R (5’-GACTACHVGGGTATCTAATCC-3’; [51]) at an original concentration of 20 µM, a sample-specific volume of DNA providing 10 ng of total DNA per sample, and PCR-grade water to complete the volume. The PCR heat profile involved an initial denaturation at 98 °C for 5 min, 25 cycles of denaturation at 98 °C for 45 s, annealing at 60 °C for 15 s, extension at 72 °C for 10 s, and a final elongation at 72 °C for 7 min. The triplicate PCR products from each sample were pooled and cleaned using magnetic beads (CleanNGS, GC Biotech) as per the manufacturer’s instructions. The cleaned PCR products were then sent to the KU Leuven Genomics Core where, after quality control, a second PCR was performed to attach Illumina adapters and indexes. This reaction used 9 µl of the first PCR product, 0.5 µl each of the forward P7 and reverse P5 primers at 5 µM, and 10 µl of Phusion high fidelity PCR master mix (Thermo Fisher Scientific), which ran at an initial denaturation of 94 °C for 30 s, followed by 15 cycles of denaturation at 94 °C for 10 s, annealing at 51 °C for 30 s, and elongation at 72 °C for 30 s, with a final elongation at 72 °C for 1 min. Subsequent quality control confirmed proper adapter attachment and an equimolar library was prepared. Given the high number of samples, sequencing was conducted in two separate runs performed on the Illumina MiSeq v3 with a 14 pM load and aimed at 20% PhiX spike-in to improve library diversity and increase sequence quality, using the 600-cycle kit for 2 × 300 bp reads. Sample distribution per run is listed in Suppl. Table 1.

#### Mock communities

Bacterial mock communities were used as positive controls with known community composition to evaluate the success of our metabarcoding experiment, determine the appropriate filtering threshold for the dataset, and the potential for DNA cross-contamination. The first Illumina sequencing run included a 10 Strain Even Mix Genomic Material MSA-1000™(ATCC) as reported and analyzed in [48] to ascertain a Relative Read Abundance (RRA) threshold in line with Reitmeier et al. [52]. Such a RRA with the help of mock communities allows for accurate filtering thresholds to improve the reproducibility of microbiome studies [52].

The second run included the ZymoBIOMICS™ Microbial Community DNA Standard I and the ZymoBIOMICS™ Microbial Community DNA Standard II (Log Distribution). Microbial Community DNA Standard I content followed by the theoretical composition of Genomic DNA (%) and 16S only (%): *Pseudomonas aeruginosa* (12, 4.2), *Escherichia coli* (12, 10.1), *Salmonella enterica* (12, 10.4), *Lactobacillus fermentum* (12, 18.4), *Enterococcus faecalis* (12, 9.9), *Staphylococcus aureus* (12, 15.5), *Listeria monocytogenes* (12, 14.1), *Bacillus subtilis* (12, 17.4), *Saccharomyces cerevisiae* (2, NA) and *Cryptococcus neoformans* (2, NA). Microbial Community DNA Standard II content followed the theoretical composition of Genomic DNA (%) and 16S only (%): *Listeria monocytogenes* (89.1, 95.9), *Pseudomonas aeruginosa* (8.9, 2.8), *Bacillus subtilis* (0.89, 1.2), *Saccharomyces cerevisiae* (0.89, NA), *Escherichia coli* (0.089, 0.069), *Salmonella enterica* (0.089, 0.07), *Lactobacillus fermentum* (0.0089, 0.012), *Enterococcus faecalis* (0.00089, 0.00067), *Cryptococcus neoformans* (0.00089, NA) and *Staphylococcus aureus* (0.000089, 0.0001).

#### Bacterial load

Quantitative PCRs (qPCRs) were used to evaluate the bacterial load in each sample, which subsequently allowed us to estimate the absolute abundance of the bacterial strains characterized through 16S rRNA gene metabarcoding [53]. qPCR reactions were run on the LightCycler^®^ 480 (Roche). Each reaction consisted of 10 µl of LightCycler^®^ 480 SYBR Green I Master (Life Science), 0.75 µl of the 16S rRNA primer 341F (5’-CCTACGGGNGGCWGCAG-3’) at 20 µM, 0.75 µl of the 16S rRNA primer 805R (5’-GACTACHVGGGTATCTAATCC-3’) at 20 µM and 1 µl of DNA (concentration 10 ng /µl) and 7.5 µl of sterile water to complete the volume to 20 µl. The temperature cycle was as follows, 5 min of initial denaturation at 95 °C, followed by 50 cycles of 45 s at 95 °C, 15 s at 60 °C and 10 s at 72 °C. Each sample was done in triplicate. The Femto™ Bacterial DNA Quantification Kit (Detection range of 20 fg-20 ng in seven steps) was used as a reference to quantify bacterial load through qPCR. This kit is based on a purified form of *E. coli* strain JM109. The melting and amplification curves were calculated through the LightCycler^®^ 480 software (v. 1.5.1). To verify the absence of primer dimers we confirmed the presence of a single peak in the melting curve. The amplification curve was then used to calculate the mean and standard deviation for both the concentration and the Ct values. Bacterial load as determined by qPCR was compared across infection status through an independent t-test if normality was met, if not, comparisons were made through the Wilcoxon rank sum test both contained in the stats package (v. 4.2.2 [54]). , .

### Processing of sequencing data

Each Illumina MiSeq run leads to unique profiles in sequencing error. Therefore, the samples for the two different MiSeq runs were processed separately until the generation of ASV tables. All steps were done according to the pipeline reported by Schols et al. [48] which was adapted from Janssens et al. [55] using the QIIME2 pipeline v2022.2 [56] on the VSC (Flemish Supercomputer Center). MultiQC was used to inspect the quality profiles of the raw reads of both runs [57]. Respectively, 37% and 12% of reads were attributed to PhiX during the first and second run. The first and second run resulted in 23,654,898 and 27,895,972 reads passing the sequencing company’s first filtering step, respectively. Of these reads, 13,643,364 and 5,825,424 were attributed to the samples of this study (19,468,788 total reads). Reads of both runs were front-end trimmed (15 bp), forward reads truncated to 280 bp and reverse reads to 240 bp with a minimal overlap of 30 bp between both reads to obtain paired-end sequences which were filtered, denoised, dereplicated and chimeras removed through the denoise function. Truncation length differed between forward and reverse reads, as reverse reads dropped faster in quality scores. Respectively, 4,021,448 (mean = 58.6%, sd = 4.7%) and 1,157,980 (mean = 45.8%, sd = 4.4%) of reads were maintained after cleaning for the first and second run.

The resulting representative sequences and data tables of both runs were then merged through the QIIME2 feature-table merge function. A total of 5,923 ASVs remained with an average length of 424 bp (sd = 11 bp). The Silva 138 SSU Ref NR 99 database [58] through a naive Bayesian classifier was used to assign taxonomy to the ASVs. The V3-V4 region sequences of this naive Bayesian classifier were obtained through the feature classifier extract reads function based on the 341 F and 805R primers with a truncation length of 465 bp. Finally, the rooted tree was generated from the representative sequences using the phylogeny align-to-tree-mafft-fasttree function. The features, taxonomy and tree files were then exported and merged with metadata to generate a phyloseq object through the qza_to_phyloseq function of the qiime2R package [59] in RStudio (v. 2022.12.0) on a Windows machine. Mock communities were analyzed separately (see next section). Contaminant ASVs were detected and removed through the Decontam package (v. 1.18.0 [60]), , using the prevalence method with a 0.05 threshold, based on two PCR negatives and one extraction negative. As a result, 25 ASVs (0.4% of the total ASVs) were classified as contaminants and removed from the dataset. Next, 179 chloroplast and mitochondrial and zero non-prokaryotic ASVs were removed and taxonomic information was corrected according to Janssens et al. [55]. 2,546 low-abundance ASVs with less than four reads per sample were removed from the dataset (See section below ‘Mock communities’ for the justification and detailing of this threshold). Subsequently, a rarefaction curve was constructed through the Vegan package (v. 2.6.4 [61]), . The dataset was rarefied 100 times through the phyloseq package (v. 1.42.0 [62]), to a fixed depth of 10,417 reads using incrementally increasing random seeds, which started at 711, and averaged the resulting OTU tables to account for stochasticity in subsampling and minimize biases introduced by single rarefaction events. As a result, 27 ASVs were removed from the dataset as well as 15 samples including the two PCR negatives, the extraction negative and three of the four mock communities. The remaining nine removed samples all originate from the second sub-experiment with various parasite infections (all originating from the second run): five Sr, three SmBRE, and two SmLE. The fourth mock sample was manually removed from the dataset, leading to the removal of 15 additional ASVs.

### Statistical analyses of snail microbiome communities

Alpha diversity was assessed using Shannon and Faith’s phylogenetic diversity indices, respectively, computed with the estimate_richness function from the phyloseq package and the pd function from the picante package (v. 1.8.2., [63]). To analyze the impact of treatment on alpha diversity, a generalized linear model was employed using the stats package. StepAIC from the MASS package (v. 7.3–58.1 [64]). , was used to perform backward selection on the global model. The model with the lowest AIC value was used in all instances except for the second research questions “What is the effect of parasite development on the snail microbiome?” whereby the null model had a slightly lower AIC value (355 vs. 363) yet we opted to investigate the interactive effect of days post-exposure and infection status. Normality was assessed with the shapiro.test function from the stats package before and after transformation. If normality was violated we assessed the skewness of the dataset through the moments package (v. 0.14.1 [65]). , and chose either log [log10(max(x + 1) - x)], square root [sqrt(max(x + 1) - x)] or inverse [1/(max(x + 1) - x)] transformation depending on what was most appropriate for each dataset. Heteroscedasticity was tested for using the leveneTest function of the car package (v. 3.1-3 [66]), and if present, accounted for through the argument white.adjust = TRUE. Outliers and influential observations were examined using the outliertest and cooks.distance functions from the car and stats packages, respectively. Detected outliers with a Bonferroni corrected *p*-value below 0.05 were removed from the dataset. Statistical significance was evaluated using a Type III ANOVA test from the car package. Overdispersion was assessed through the check_overdispersion function of the performance package (v. 0.11.0 [67]). , . When applicable a pairwise comparison was made for each level through the lsmeans package with a Tukey correction for multiple testing (v. 2.30.0 [68]), . The McFadden pseudo-R² value was calculated using the pR2 function from the pscl package (v. 1.5.9 [69]). , when assessing the effect of the proportion of reads attributed to a schistosome infection on the Shannon diversity in each sample. Detailed statistical approaches are listed in Table 1.

**Table 1:** Statistical modeling. Response: lists the response variable and any required transformation. Predictors: predictor variables used as input for the models. #N: sample size. Figures: reference to the figure that show the associated output

Question	Statistical approach	Response	Predictors	#*N*	Figures
Do miracidia-exposed snails have a different microbiome from control snails? (Fig. 1)	GLM	Log Shannon & Faiths PD	Parasite exposure (1mir, 10mir, co-expSmLE-SmBRE, co-expSmLE-Sr, control)	212	Suppl. Figure 2
What is the effect of parasite development on the snail microbiome? (Fig. 1B)	GLM	Shannon	Days post-exposure (6, 10, 16, 20, 30, 40), infection status (yes, no), days post-exposure: infection status (interaction)	60	Suppl. Figure 3
	GLM	Faiths PD	Idem	60	Figure 2
	RDA	Bray-Curtis dissimilarity, weighted Unifrac and unweighted Unifrac distance	Idem	61	Figure 2, Suppl. Figure 7
	GLM	Shannon	Days post-exposure, proportion of reads attributed to trematodes, their interaction	60 & 9	Suppl. Figure 4
How do different parasite species affect the snail microbiome? (Fig. 1C)	GLM	Inverse Shannon	Parasite exposure (SmLE, SmBRE, Sr), parasite infection (uninfected, SmLE, SmBRE, Sr), infection status (yes, no)	79	Figure 3
	GLM	Square root Faiths PD	Parasite exposure (SmLE, SmBRE, Sr), parasite infection (uninfected, SmLE, SmBRE, Sr), infection status (yes, no)	79	Suppl. Figure 9
	RDA	Bray-Curtis dissimilarity, weighted Unifrac and unweighted Unifrac distance	Idem	79	Figure 3, Suppl. Figure 10
How do parasite species under co-infection affect the snail microbiome? (Fig. 1D&E)	GLM	Log Shannon	Infection status (uninfected, SmLE, SmBRE, Sr, Sr + SmLE, SmBRE + SmLE), days post-exposure (2, 10, 40), parasite exposure (co-expSmLE-SmBRE, co-expSmLE-Sr)	62	Suppl. Figure 12
	GLM	Faiths PD	Idem	62	Figure 4
	RDA	Bray-Curtis dissimilarity, weighted Unifrac and unweighted Unifrac distance	Idem	62	Figure 4, Suppl. Figure 13

Beta diversity was explored through the Bray Curtis dissimilarity measure, visualized using NMDS and RDA via the ordinate function from the phyloseq package and the capscale function from the vegan package. To assess consistency across different diversity measures, the RDA analyses were then repeated using the unweighted and weighted Unifrac distance. If the phylogenetic tree ended up with an edge matrix with an odd number of rows we transformed all multichotomies into a series of dichotomies using the multi2di function of the ape package (v. 5.6-2 [70]). , whereafter the tree was rerooted using the root_phyloseq_tree function of the QsRutils package (v. 0.1.5 [71]). , . The ordistep function from the vegan package was used for automatic stepwise model building after which the anova function was called to assess model significance (Table 1). Collinearity was assessed through the variance inflation factor obtained through the vif.acc function from the vegan package. Subsequently, the within-group Bray-Curtis dissimilarity was investigated based on an adapted version of the micrUBIfuns function (v. 0.1.0 [72]). , and differences in means between levels tested with the Wilcoxon rank sum test with a Bonferroni correction for multiple testing.

In line with Lloyd-Price et al. [73] samples highly divergent from the reference dataset were identified based on the 90th percentile threshold. Core microbiome analysis using a detection threshold of 0.01 − 0.001 with a prevalence allowing up to one missing sample was done based on the microbiome package (v. 1.20.0 [74]), while adhering to the guidelines of Neu, Allen and Roy [75]. The resulting core microbiome results are reported as the mean, minimum, and maximum read proportion per sample. Venn diagrams were constructed based on unique ASV names through the Bioinformatics and Evolutionary Genomics website of Ghent University (https://bioinformatics.psb.ugent.be/webtools/Venn/). Differential abundance analysis based on the negative binomial distribution was performed through the DESeq2 package (v. 1.38.3 [76]). , .

### Mock communities

The ATCC mock communities have been thoroughly analyzed and reported by Schols et al. [48], resulting in the most appropriate RRA threshold of 0.5%. The ZymoBIOMICS™ Microbial Community DNA Standard with normal distribution failed (189 reads after filtering). The log-distributed mock community was successfully sequenced and did not contain any contaminating ASV while detecting almost all expected bacterial species, except for the three rarest bacterial taxa *Lactobacillus fermentum*, *Enterococcus faecalis*, and *Staphylococcus aureus* and not detecting the two fungi. The three taxa not detected in the log-distributed mock community were detected in the few reads of the normal distribution mock community. *Escherichia coli* and *Salmonella enterica* (respectively, 0.089% Genomic DNA and 0.069% 16S only, and 0.089% Genomic DNA and 0.07% 16S only) were the detection limit of our pipeline with four and seven reads, respectively. Hence a RRA threshold suitable to exclude contaminants was more difficult to ascertain. Therefore, ASVs with less than four reads per sample were set to zero for that sample as it falls below the detection limit of our tool, rather than a RRA threshold for filtering. This is in line with the removal of single-, double-, and tripletons from a dataset, yet at the sample level, as they are most likely erroneous [77, 78].

## Results

### Bacterial patterns

#### Comparing miracidia exposed snail samples to control snails

To assess whether parasite exposure affected the snail microbiome, control (Fig. 1A) and exposed (Fig. 1B-E) snails were compared. The Shannon diversity required log-transformation to meet normality and a heteroscedasticity correction (untransformed: Suppl. Figure 2A & transformed: Suppl. Figure 2B). Pairwise comparison revealed a significant difference between both control sample groups, and between each control sample group and all parasite exposure groups (*p* < 0.03) except between control snails at day 0 and 10 miracidia SmBRE exposed snails (*p* = 0.62). Only 10 miracidia SmBRE exposed snails and those co-exposed to SmLE and Sr (*p* < 0.03) differed significantly from each other across the parasite exposure groups. The Faith’s phylogenetic diversity did not require transformation to meet normality but also required accounting for heteroscedasticity. It showed similar patterns with none of the parasite exposure groups differing significantly from each other, and all control snails differing significantly from parasite exposed groups except control snails at day 0 both with 10 miracidia SmBRE (*p* = 0.28) and 10 miracidia Sr exposed snails (*p* = 0.07, Suppl. Figure 2C). NMDS revealed separate clustering of unexposed snails collected at 0 days post-exposure (daysPE) for the Bray-Curtis dissimilarity measure (Suppl. Figure 2D) but not for the weighted and unweighted Unifrac distance (Suppl. Figure 2E-F). Group composition could not be assessed due to heterogeneity in the dataset (permutest; F = 3.85, *p*-value = 0.048). The ASV-level core microbiome contained a *Cloacibacterium* sp. (min = 0.0005, max.=0.74, mean = 0.14, sd = 0.14) if up to two samples were allowed to have a detection prevalence below 0.001.

#### The temporal pattern throughout schistosome development (Fig. 1B)

Two, 6, 10, 16, 20, 30 and 40 days following parasite exposure, infected and uninfected snails were compared to study the effect of parasite development on snail microbiome. When investigating the interactive effect of daysPE and infection status on the Shannon and Faith’s phylogenetic diversity metrics, normality was achieved after removing the same outlier for each metric (*N* = 1, uninfected sample at 30 daysPE). No significant effect of daysPE (Shannon: LR Chi²= 5.38, *p*-value = 0.37; Faith’s PD: LR Chi²=3.83, *p*-value = 0.57), infection status (Shannon: LR Chi²= 0.30, *p*-value = 0.59; Faith’s PD: LR Chi²=0.87, *p*-value = 0.35), or their interaction (Shannon: LR Chi²= 7.43, *p*-value = 0.19; Faith’s PD: LR Chi²=8.12, *p*-value = 0.15) was detected. However, pairwise comparisons revealed significantly higher alpha diversity (Shannon: estimate = 1.24, SE = 0.47, df = 43, t-ratio = 2.63, *p*-value = 0.01; Faith’s PD: estimate = 10.23, SE = 4.13, df = 43, t-ratio = 2.48, *p*-value = 0.02) in uninfected samples compared to infected samples at 30 daysPE (Suppl. Figure 3, Fig. 2A). Shannon diversity and Faith’s PD were not affected by the proportion of reads attributed to SmBRE across the entire experiment (Suppl. Figure 4 A&B), with the exception of 30 daysPE (Suppl. Figure 4 C&D; Shannon: LR Chi²=7.23, *p*-value = 0.007, McFadden R²=0.26; Faith’s PD: LR Chi²=8.51, *p*-value = 0.003, McFadden R²=0.12). Notably, not removing the outlier with a Bonferroni p below 0.05, causes the difference between uninfected and infected snails at 30 daysPE to become non-significant (Suppl. Figure 5; Shannon *p*-value = 0.19, Faiths PD *p*-value = 0.24). Bacterial load, measured via qPCR, was significantly affected by daysPE (LR Chi²= 26.24, *p*-value < 0.001) and infection status (LR Chi²= 8.39, *p*-value = 0.004) but not their interaction (LR Chi²= 10.39, *p*-value = 0.06). Specifically, it was lower in infected compared to uninfected samples at 16 daysPE (Suppl. Figure 6; estimate = 0.67, SE = 0.23, df = 44, t-ratio = 2.98, *p*-value = 0.005).

Ordistep model selection for RDA indicated that infection status did not affect the bacterial community (F = 1.03, *p*-value = 0.40), while daysPE did have a significant effect (Fig. 2B; F = 6.01, *p*-value < 0.001) when considering the Bray–Curtis dissimilarity. Weighted and unweighted Unifrac distances showed an identical pattern (Suppl. Figure 7). The within-group Bray–Curtis dissimilarity differed significantly between infected and uninfected snails at six and ten daysPE, respectively (Fig. 2C). The core microbiome at ASV-level across uninfected samples contained an *Uliginosibacterium* sp. and a Comamonadaceae sp. at a detection threshold of 0.001 (min = 0.003, max.=0.176, mean = 0.062, sd = 0.047). The core microbiome at ASV-level across infected samples consisted of eight ASVs: one *Blastopirellula* spp., two *Uliginosibacterium* sp., a *Cloacibacterium* sp., a *Mycoplasma* sp., a Phycisphaeraceae sp., a Isosphaeraceae sp., and a Comamonadaceae sp. at a detection threshold of 0.001 (min = 0.026, max.=0.634, mean = 0.294, sd = 0.184). At 30 daysPE ten ASVs were present in all uninfected samples: two *Mycoplasma* spp., three Comamonadaceae spp., a *Chryseobacterium* sp., a *Pseudomonas* sp., a Chlamydiales sp., a Phycisphaeraceae sp., and a Caldilineaceae sp. (min = 0.068, max.=0.288, mean = 0.182, sd = 0.080). In contrast, 16 ASVs were present in all infected samples: two *Blastopirellula* spp., an *Uliginosibacterium* sp., a *Cloacibacterium* sp., two *Mycoplasma* spp., a *Fimbriiglobus* sp., a Bacilli sp., a Phycisphaeraceae sp., two *Pirellula* sp., an Isosphaeraceae sp., and four Comamonadaceae sp. (min = 0.224, max.=0.712, mean = 0.477, sd = 0.183). At the genus level *Cerasiococcus* and *Hypomicrobium* were found more abundantly in uninfected compared to infected samples (Log2fold change of 2.74 and 1.86, resp.; Padj < 0.05). Further investigation of this pattern through a heatmap at ASV level did not reveal a clear pattern across daysPE (Suppl. Figure 8).

#### The effect of parasite population and species in single infections on the microbiome (Fig. 1C)

To understand how different parasite species and populations affect the snail microbiome, snails exposed to three different parasites were studied 40 days after parasite exposure. For the alpha diversity, the Shannon diversity metric required inverse transformation to meet normality. Only parasite infection (uninfected, or established infections of SmLE, SmBRE and Sr) was included through stepwise model selection. The infection status significantly influenced the inverse Shannon diversity after correcting for heteroscedasticity and removing one outlier SmBRE infected sample (F = 9.29, *p*-value < 0.001). A pairwise comparison revealed a significantly lower inverse Shannon diversity of snails infected by SmLE or Sr when compared to SmBRE (Fig. 3A, Suppl. Figure 9A; estimate=-0.16, SE = 0.05, z-value=-2.96, *p*-value = 0.02; estimate=-0.16, SE = 0.05, z-value=-3.22, *p*-value = 0.006, resp.) and compared to uninfected snails (estimate=-0.13, SE = 0.04, z-value=-3.48, *p*-value = 0.003; estimate=-0.13, SE = 0.03, z-value=-4.14, *p*-value < 0.001, resp.). Generally similar patterns were also noted for the inverse Faith’s phylogenetic diversity metric after removing the same outlier. Stepwise selection also only kept the parasite infection in the model (Suppl. Figure 9B&C; LR Chi²= 23.34, *p*-value < 0.001). Only SmLE-infected snails remained with a significantly lower inverse transformed Faith’s phylogenetic diversity from SmBRE-infected snails (estimate = 0.99, SE = 0.21, df = 65, t-ratio = 4.81, *p*-value = 0.0001). Bacterial load, measured via qPCR, was not affected by parasite infection status (LR Chi²= 4.07, *p*-value = 0.25).

Ordistep model selection for beta diversity selected the additive effect of infection status (infected or uninfected) and parasite exposure (exposure to SmLE, SmBRE or Sr) when considering the Bray–Curtis dissimilarity. The entire model was significant (Fig. 3B; F = 9.55, *p*-value < 0.001) as well as infection status (F = 3.24, *p*-value = 0.007) and parasite exposure (F = 12.24, *p*-value < 0.001). The same pattern is seen for the weighted and unweighted Unifrac distance (Suppl. Figure 10), except for a significant effect of the interaction term in the former (F = 2.16, *p*-value = 0.027). The within-group Bray–Curtis dissimilarity differed significantly between all parasite infections and when compared to uninfected samples, except for snails infected with *S. rodhaini* which were not significantly differing from uninfected snails and SmLE-infected snails compared to SmBRE-infected snails (Fig. 3C). The core microbiome at ASV-level across all samples consisted of five ASVs: a Phycisphaeraceae sp., a Planctomycetes sp., a *Uliginosibacterium* sp., a *Cloacibacterium* sp., and a Comamonadaceae sp. (min = 0.019, max.=0.422, mean = 0.246, sd = 0.099). An additional 52 ASVs made up the core microbiome of uninfected samples (min = 0.647, max.=0.783, mean = 0.733, sd = 0.041). When looking at the core microbiome of various infections (Sr, SmBRE, and SmLE) 21, 15, and 7 ASVs were respectively detected (resp., min = 0.478, max.=0.724, mean = 0.632, sd = 0.073; min = 0.036, max.=0.614, mean = 0.401, sd = 0.154; min = 0.147, max.=0.822, mean = 0.441, sd = 0.145; Suppl. Figure 11).

#### The effect of parasite population and species under co-infection on the Microbiome (Fig. 1D-E)

**Fig. 4:**
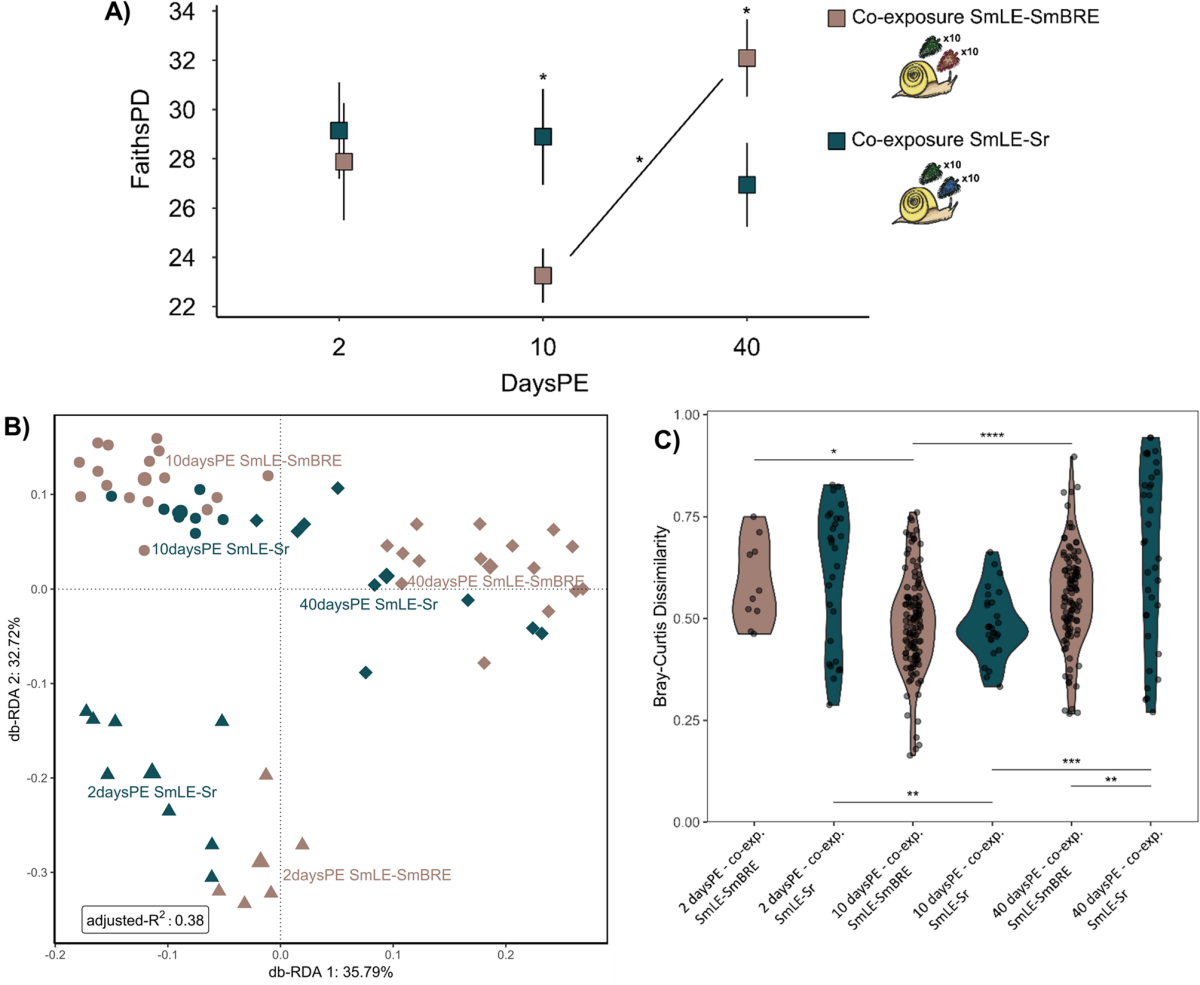
The bacterial aspect of the microbiome during the two co-infection experiments (SmLE & SmBRE, and SmLE & Sr; as indicated by the color code) across 2, 10, and 40 daysPE (irrespective of final infection outcome). **A**) The alpha diversity as calculated through the faith’s phylogenetic diversity metric, based on mean values with error bars representing a single standard deviation. The line represents the pairwise comparison between 10 and 40 daysPE for the co-exposure of SmLE-SmBRE. **B**) Bray-Curtis dissimilarity measure across the different days post exposure (indicated by the different shapes with the centroid indicated by the largest symbol) as shown by a RDA plot. The RDA considers the co-infection experiment, days post exposure and their interaction. Ordistep excluded the infection diagnosis. **C**) The within-group Bray–Curtis dissimilarity with labels on the x-axis indicating the days post exposure (first number) followed by co-infection experiment (‘co-exp. SmLE-Sr’ and ‘co-exp. SmLE-SmBRE’). Significance: <0.05=‘*’, < 0.01=‘**’, < 0.001=‘***’, < 0.0001=‘****’. Sample counts per condition can be found in Fig. 1 and Suppl. Table 1

Snails of two co-exposure conditions were compared during infection development to understand how co-infections shape the snail microbiome. The additive effect of daysPE (2, 10, and 40), parasite exposure (co-exposure to either SmLE and SmBRE, or SmLE and Sr) and their interaction were selected by the model when considering the Shannon diversity measure. No significant effect of daysPE (LR Chi²= 1.10, *p*-value = 0.58) and parasite exposure (LR Chi²= 2.02, *p*-value = 0.15) was detected yet their interaction was significant (LR Chi²= 10.04, *p*-value = 0.007). Pairwise comparisons revealed a significantly higher Shannon diversity of snails co-exposed to SmLE and Sr compared to snails co-exposed to SmLE and SmBRE at 40 daysPE (Suppl. Figure 12; estimate = 0.168, SE = 0.076, df = 55, t-ratio = 2.19, *p*-value = 0.03). No significant change occurred throughout the experiment in snails co-exposed to SmLE and Sr while the microbiome changed significantly when comparing ten and 40 days post-exposure (estimate=-0.18, SE = 0.065, df = 55, t-ratio=-2.80, *p*-value = 0.02). A similar pattern is noted for the Faith’s phylogenetic diversity metric. Also, here the interaction between daysPE and parasite exposure was significant (LR Chi²= 11.80, *p*-value = 0.003) while the individual variables were not. Pairwise comparisons revealed a significantly higher diversity in snails co-exposed to SmLE and Sr compared to SmLE and SmBRE at 10 daysPE (Fig. 4A; estimate=-0.227, SE = 0.082, df = 55, t-ratio=-2.78, *p*-value = 0.008) while the opposite is true at 40 daysPE (Fig. 4A; estimate = 0.164, SE = 0.079, df = 55, t-ratio = 2.059, *p*-value = 0.04). Only snails co-exposed to SmLE and SmBRE differed significantly between ten and 40 daysPE in either co-infection experiment (Fig. 4A; estimate=-0.32, SE = 0.07, df = 55, t-ratio=-4.738, *p*-value < 0.001).

RDA indicated that daysPE (F = 10.88, *p*-value < 0.001), parasite exposure (F = 4.41, *p*-value < 0.001) and their interaction (F = 4.28, *p*-value < 0.001) significantly affected the bacterial community (Fig. 4B). The same pattern is seen for the weighted and unweighted Unifrac distance (Suppl. Figure 13). The within-group Bray–Curtis dissimilarity differed significantly between two and ten, and ten and 40 daysPE for each of the parasite exposures separately but only differed significantly at 40 daysPE between the two experimental conditions (Fig. 4C). This pattern was not correlated with established infections. The core microbiome at ASV-level across both co-infection experiments consisted of a *Cloacibacterium* sp. and a Comamonadaceae sp. at a detection threshold of 0.001 (min = 0.002, max = 0.749, mean = 0.168, sd = 0.145). The SmLE/SmBRE co-infection experiment core microbiome is further supplemented with an *Uliginosibacterium* sp. and an *Aquabacterium* sp. (min = 0.012, max = 0.559, mean = 0.205, sd = 0.145) while that of the Sr/SmLE co-infection experiment was supplemented by a Planctomycetes sp. (min = 0.005, max = 0.750, mean = 0.200, sd = 0.174). Following DeSeq2 analysis, heatmap plotting indicates stronger clustering per experiment for ten and 40 daysPE compared to two daysPE (Suppl. Figure 14). Moreover, SmLE and SmBRE co-exposed snails showed increased read abundance of the genera *Achromobacter*, *Stenotrophomonas*, *Pedospaeraceae*, and *Thermomonas* (Suppl. Figure 15). In contrast, SmLE and Sr co-exposed snails showed increased read abundance of the genus *Schlesneria*, and to a lesser extent *Mycobacterium* sp.

## Discussion

In this study, we reveal that (i) the microbiome of *Biomphalaria glabrata* exhibits a transient but significant reduction in bacterial load and alpha diversity around 16 and 30 days post-infection, respectively; (ii) high-shedder *S. mansoni* (SmLE) induce stronger microbiome disruption than low-shedder *S. mansoni* (SmBRE) or *S. rodhaini*; and (iii) under co-infection, *S. rodhaini* appears to buffer the dysbiotic effect of SmLE.

The rise of metabarcoding tools capable of characterizing (co-)infecting parasites within snail samples (such as [36, 79]) has set the stage for snail-parasitome-microbiome interaction studies. These improved infection characterization tools combined with a different experimental approach enable us to build on the valuable insights of Portet et al. [7]. Briefly, these authors revealed a non-reciprocal effect of snails on the bacterial profile of the water, a temporal change in the snail associated microbiome across alpha and beta diversity metrics, a more pronounced effect of re-exposure (i.e., parasite challenge of already exposed snails) vs. primary exposure irrespective of sympatric or allopatric *B. glabrata-S. mansoni* combinations on alpha diversity metrics, and an effect through time, (re-)exposure and sympatric-allopatric combinations at the community level.

Despite an overlap in the general idea behind the experiment, several notable differences were exploited in our experimental setup compared to Portet et al. [7]. First, an increased sampling effort through time focused on parasite development during the first part of our experiment (i.e., snail collection at 2, 6, 10, 16, 20, 30 and 40 days post-exposure vs. 1, 4 and 25). Second, also during this part of the experiment, snails were exposed to a single miracidium (1 vs. 10), providing a higher number of uninfected snails for direct comparison as they were exposed to and reared under the same conditions in the same aquarium. Third, co-infections were obtained through simultaneous not sequential parasite exposure of snails. Fourth, we worked with a different population of *B. glabrata* (BgGUA2 vs. BgGUA, BgBAR, BgBRE, and BgBS90). Fifth, we included *S. rodhaini*, a closely related schistosome to *S. mansoni*. Finally, across all experiments we determined the infection status through molecular diagnostic tools, allowing a comparison at the infection level and not only parasite exposure like Portet et al. [7].

The use of SmBRE in the mono-miracidial infection time series and SmLE–Sr in the co-infection sub-experiment reflects complementary design choices: the gradual development of SmBRE enabled sensitive, time-resolved tracking of microbiome shifts, whereas the SmLE–Sr combination provided the clearest test case for antagonistic exclusion and microbial community effects [36, 40, 44]. Together, these sub-experiments highlight different yet complementary aspects of schistosome–snail–microbiome interactions.

### The temporal pattern throughout schistosome development (Fig. 1B) – the onset of shedding an intricate parasite-snail interaction?

To examine the effect of key parasite developmental stages on the snail microbiome, snails were individually exposed to a single SmBRE miracidium and sacrificed at 2, 6, 10, 16, 20, 30, and 40 days post-exposure. The alpha diversity significantly differed between infected and uninfected snails but only at 30 days post-miracidium exposure and with removal of an outlier (Fig. 2, Suppl. Figure 4B). We hypothesize that this temporary significant difference in alpha diversity may be attributed to the onset of cercarial shedding. Using the formula from Pflüger [45] “268/(Temperature-14.2) = time until onset of shedding”, with our rearing temperature of 25 °C, the estimated onset of shedding is around 25 days post-exposure. However, Theron [47] noted the onset of shedding at 33 days after miracidial exposure at 26 °C. Therefore, we estimate that snails began shedding in our experiment around the 30 day mark. Around 30 days post-exposure, schistosome biomass [46] and read count [36] level off, potentially coinciding with the onset of shedding. Le Clec’h et al. [40] and Théron [47] revealed, for SmBRE and an unknown *S. mansoni* population from Brazil, respectively, that the number of released cercariae peak in the first week after the onset of shedding after which the numbers reduce. This pattern might explain why alpha diversity differs between infected and uninfected snails at 30 days post-exposure but not at 40 days. Similarly, following a drop in glucose concentration between 28 and 35 days post-exposure, glucose levels increase again at 42 days post-exposure [35], potentially also influencing the observed pattern.

For now, it remains unclear whether the drop in alpha diversity is (1) directly linked to mechanical damage and biochemical means required for the onset of shedding [31, 35, 80], (2) a consequence of the release of biomass and therefore reduced bacterial habitat, or (3) resulting from a relatively lower bacterial DNA template available for PCR amplification due to the increase in parasite DNA [36]. Cercariae bursting through the mantle epithelium cause severe damage to snail tissue [80, 81]. Such wounds can provide an entry point for opportunistic environmental pathogens [82], and are linked to bacterial dysbiosis and an increase in potential pathogens in corrals [83, 84]. Nevertheless, no signature of a dysbiotic state was seen in this part of the dataset (Fig. 2C). Reduced bacterial DNA is unlikely to be the sole driver behind the observed pattern as the reduction in bacterial load in infected snails was nonsignificant at 30 days post-exposure (see Sect. 3.1.2. & Suppl. Figure 6). Furthermore, *B. glabrata* egg count starts to drop three to four weeks after exposure to *S. mansoni* miracidia [85, 86]. This suggests that the drop in alpha diversity may also coincide with the onset of snail castration and cercarial release. Moreover, infected samples at 16 days post-exposure did show a significantly lower bacterial load compared to their uninfected counterparts but did not show a reduction in any alpha diversity metric. This timing coincides with the release of daughter sporocysts from mother sporocysts and the onset of their migration to the gonad-digestive gland region [33, 44]. Combined, this data suggests an intricate snail-parasite-microbiome interaction during key points of parasite development and release. Additionally, the mono-miracidial exposure used in the time series—though experimentally advantageous for maximizing uninfected controls—may have led to subtler physiological and microbial responses compared to multi-miracidial exposures [34, 42], potentially limiting the detectability of microbiome shifts during infection development.

In general, the consensus is that the initial parasite-snail interplay determines infection outcome [41, 87]. Nevertheless, a later stage, when schistosome cercariae mature and emerge, is arguably equally impactful on snail physiology [31]. The data represented here suggests that microbiome-mediated schistosomiasis control in snail hosts could also attempt to target this period of parasite development. Indeed, *Wolbachia* infections in mosquitoes offer similar opportunities with severely reduced transmission to the next host by preventing infection but also by restraining the formation of the infective stage, the sporozoite [88, 89]. Tavalire et al. [34] posit a similar perspective after observing delayed shedding of up to 30 weeks in certain inbred *B. glabrata* lines with equal resistance to infection. Moreover, snail lines capable of delaying the onset of shedding, and thus castration, also increased their reproductive period thereby underlining the viability of this target for fixation in natural populations [34]. Perhaps the microbiome was an underlying factor driving this delayed shedding which may be induced artificially through microbial manipulations. To test this hypothesis and ascertain a causative relationship between the microbiome and the onset of shedding, future experiments may attempt to artificially increase the alpha diversity around the 30-day mark and study the effect on cercarial release.

### The effect of parasite population and species in isolated infections on the microbiome during the shedding period (Fig. 1C)

To assess the effect of mature infections on the snail microbiome, snails were individually exposed to 10 miracidia from each of three parasite populations and sacrificed 40 days later. High-shedder *S. mansoni* (SmLE) infections had a more profound effect on alpha diversity compared to low-shedder *S. mansoni* (SmBRE) infections (Fig. 3A, Suppl. Figure 9), which we hypothesize could be linked to the different virulence levels of both populations. The SmBRE population produces fewer cercariae, as is the case for *S. rodhaini*, while SmLE sheds eight times more cercariae and produces significantly more mother sporocysts [12, 40, 42, 90, 91], suggesting a higher impact on snail physiology and thereby the microbiome. The effect cannot be explained by reduced space for bacteria imposed by SmLE infections, as the bacterial load did not differ significantly between the established infections (see Sect. 3.1.3. bacterial load). Both *S. mansoni* populations increased dysbiotic state of infected snails during the shedding period while *S. rodhaini* did not (Fig. 3C), which might be explained by a different intra-molluscan development or species-specific adaptation to the intermediate snail host. Indeed, *S. mansoni* daughter sporocysts all move directly to the gonad-digestive gland region while part of the *S. rodhaini* daughter sporocysts remain in the head-foot region before joining the other daughter sporocysts that directly migrated to the gonad-digestive gland region [44]. Moreover, *S. mansoni* cercanogenous sporocysts (sporocysts producing cercariae) can transform to sporocystogenous sporocysts (sporocyst generating sporocysts) while no such transformations have been detected for *S. rodhaini* [44, 92]. Therefore, one hypothesis could be that these developmental variations explain why *S. mansoni* induces a dysbiotic state while *S. rodhaini* does not. Furthermore, SmLE and SmBRE infections had an *Aquabacterium* sp. as one of two core microbiome members, which was not in the core microbiome of uninfected and *S. rodhaini-*infected samples. Moreover, *Aquabacterium* spp. are described from many aquatic environments [93–95] but more importantly, do not appear to metabolize carbohydrates [95], one of the key parasite energy sources in the snail [35]. Therefore, the lack of competition with the parasite potentially explains why this bacterium became so abundant. A second hypothesis involves species-specific, and not population-specific, snail-parasite interactions whereby SmLE and SmBRE naturally infect *B. glabrata* while Sr infects *B. pfeifferi*. Since the dysbiotic state is frequently linked to disease [22–24], this data suggests that parasites pre-adapted to the same snail species might be more virulent and increase the chance for dysbiotic state and potentially increase resource acquisition from the snail host. Disturbing the microbiome might prove beneficial for the parasite as it could disrupt the host’s immune response [96]. Indeed, dysbiosis is induced by nematode infection in susceptible but not resistant gastropod species [97]. In contrast to trematodes, however, this nematode can feed on dead snails, mitigating the loss of fitness incurred by killing its host and potentially exacerbating dysbiotic patterns. Indeed, excessive disruption might increase host mortality and thereby also the trematode’s, as shown by the increased snail mortality due to SmLE infections compared to SmBRE [40]. Contrary to multi-miracidial infections, mono-miracidial infections with SmBRE do not cause dysbiosis (Fig. 2C). Combined, the presented data suggests that the bacterial profile depends on parasite virulence, infection load, and parasite adaptation to snails at the species level.

### The effect of parasite population and species under co-infection on the microbiome (Fig. 1D & E)

To assess the effect of co-infections on the snail microbiome, snails were individually exposed to 10 miracidia from each parasite population (SmLE-Sr or SmLE-SmBRE) and sampled at 2, 10, and 40 days post-exposure. Model selection indicated no effect of infection status (infected yes/no) and parasite infection (uninfected, SmLE, Sr, SmBRE, SmLE-Sr, or SmLE-SmBRE) on the snail microbiome. Instead, it suggests a change through time and an effect of parasite exposure (Fig. 4A, Suppl. Figure 12).

SmLE is known to outcompete SmBRE and *S. rodhaini* under co-infection [12, 36], suggesting also a dominant effect of SmLE on the microbiome. However, this pattern is only visible under co-infection with SmBRE but not with *S. rodhaini* at 40 days post-exposure, despite outcompeting both parasites as witnessed by metabarcoded snail infections [36], revealing that this effect is not due to co-infection in itself but due to a direct or indirect parasite-parasite interaction. Combined, these results suggest that SmBRE did not interfere with the impact of SmLE on the snail’s bacterial profile, while *S. rodhaini* appears to have a stabilizing effect on the snail’s microbiome despite eventually being outcompeted by SmLE [36]. Indeed, co-exposure of *S. mansoni* and *S. rodhaini*, leading to both single- and co-infections, reduced cercarial output for both parasites [12], supporting an effect of outcompeted *S. rodhaini* on the *S. mansoni-B. glabrata*-microbiome interaction. Notably, the metabarcoding tool used to determine the infection status of these snail samples was specifically designed to only detect infections older than two days, in order to avoid false positives (i.e., detecting aborted infections) [36]. Hence, *S. rodhaini* infections may still have persisted as a single dormant sporocyst, that could not be picked up by our tool. Nevertheless, the data discussed here suggests that outcompeted parasite populations can affect the tripartite interaction between intermediate hosts, their microbiome, and parasites, further complicating this field of research. Indeed, uninfected but exposed snails show a similar increase in reproductive output as infected snails before the onset of castration [98], revealing an intricate effect of parasite exposure on snail biology possibly extending to the snail microbiome.

### General patterns

Bacterial communities varied through time, independently of infection status (Figs. 2B and 4B). Since the infection experiments were conducted with same-age snail cohorts, this result indicates a possible change in the microbiome throughout snail development. Indeed, Chen et al. [99] report a significantly different microbiome when comparing juvenile and adult apple snails (*Pomacea canaliculate*). Moreover, a changing microbiome during host ageing is broadly acknowledged in several organisms like humans, fish, flies and mice (see the reviews of Aleman [100] and Stock [20]). However, the bacterial communities did not follow a directional shift through time, suggesting that another factor might be at the basis of this pattern.

*Aeromonas* and *Cloacibacterium* have been described as core members of the gut microbiome of several gastropod species, including *B. glabrata* [101]. The relative abundance of these taxa per sample ranged between 0 and 50% with means around 10–20% of the read count [101]. Similarly, we detected a *Cloacibacterium* sp. as a sole member of the core microbiome with a mean relative read abundance of 14% (sd = 14%), indicating that the proportion of *Cloacibacterium* sp. in the gut microbiome is similar to that of the whole snail organism. However, we did not detect any *Aeromonas* sp. as core members in any of the sub-experiments nor in the entire experiment, suggesting that this taxon might be less ubiquitously present in freshwater snails than previously anticipated [101]. Uninfected samples showed less diverse core microbiomes compared to infected samples throughout most of the experiment. The established infections of each parasite population showed a unique core microbiome, supporting earlier evidence of a species-specific influence of trematode parasites on the microbiome [102]. Notably, part of our experiments reveal a higher impact of actual infection status rather than initial parasite exposure on the microbiome. This reveals a critical gap in the current understanding, as most previous studies primarily focused on the effects of parasite exposure on the snail’s microbiome without considering the infection status. Therefore, we propose infection diagnostic tools, to characterize infection establishment, to become an integral part of future lab-based parasite exposure experiments.

### Limitations

The genetic diversity of the *B. glabrata* population (BgGUA2) used in this study, as previously reported by Hammoud et al. [36], could contribute to within-population variation in susceptibility to schistosome infection [103–105]. While this reflects natural heterogeneity and increases ecological relevance, it also introduces additional within-group variability which could have affected our results. However, infection status was determined post hoc using high-resolution metabarcoding rather than inferred from exposure alone, mitigating confounding effects of host genetic variation in susceptibility. Nonetheless, broader sampling across and within snail populations would help assess the influence of host genetic background on the observed patterns.

As we worked with whole snail DNA extracts (excl. the shell) to monitor the entire holobiont, no information at tissue level has been obtained. Investigating the microbial patterns at tissue level could not only allow a comparative analysis at different parasite migration and development stages [35, 44, 92, 106], but it could also increase the sensitivity of the analysis and perhaps increase the detection of rare taxa, which can be linked to important metabolic functions [107]. Transient microbes from the salad diet likely contributed to the dataset [108], further confounding rare taxa. However, since all snails were fed *ad libitum* and were not starved before extraction, any potential bias should be consistent across samples. Moreover, starvation has been shown to have little effect on snail gut microbiota [109, 110], and in this context would likely have introduced additional stress and mortality in infected snails [111].

Although valuable insights can be drawn from the reported dataset, experimental artefacts due to small sample sizes for some of the experiments and single aquaria per parasite exposure condition may be a problem for the interpretation of our results [112]. Ideally, snails should be kept in individual aquaria to obtain fully independent replicates and a sufficient number of replicates per treatment should be obtained [112, 113]. However, Portet et al. [7] showed that rearing conditions do not affect the microbiome, while our dataset did not reveal bacterial profiles depending on the sub experiment, and thereby the aquarium (see Sect. 3.1.1, Suppl. Figure 2D-F), suggests a limited effect of experimental artefacts for the current study. Furthermore, the common garden of snails of the same treatment does allow direct comparison between infected and uninfected samples.

### Conclusion

The data represented in this study revealed a complex interplay between schistosome biology and microbiome profiles in the snail *B. glabrata*. We revealed an intricate snail-parasite-microbiome interaction during key points of parasite development and a stabilizing effect of outcompeted parasites on this interaction. Most importantly, we show that exposed but uninfected snails have a distinctly different microbiome from infected snails, revealing the necessity of infection diagnostic tools in future parasite exposure experiments studying the snail microbiome. Hopefully, studies like ours can contribute to solving the complex tripartite interaction puzzle and eventually lead to potential avenues for sustainable schistosomiasis control.

## Supplementary Information

Below is the link to the electronic supplementary material.

Supplementary Material 1

## Data Availability

The dataset generated and analysed during the current study is available in the European Nucleotide Archive (ENA) repository, under bioproject PRJEB77058 (https://www.ebi.ac.uk/ena/browser/view/PRJEB77058). The R-script used to analyze the sequencing data together with associated metadata and intermediate phyloseq objects are available on Github (https://github.com/RubenSchols/Schistosome-species-parasite-development-and-co-infection-combinations-determine-microbiome-dynamics).
