# Supplementary material for: Schistosome species, parasite development, and co-infection combinations determine microbiome dynamics in the snail Biomphalaria glabrata
Source: Anim Microbiome. 2025 Oct 2;7:101. doi: 10.1186/s42523-025-00471-3 (PMC12492517; doi:10.1186/s42523-025-00471-3)
Supplement: Supplementary file 1 — Supplementary Material 1 [file 42523_2025_471_MOESM1_ESM.docx]

**Appendix – Schistosome species, parasite development, and co-infection combinations determine microbiome dynamics in the snail *Biomphalaria glabrata***

Suppl. Table 1: Sample counts per experimental condition. Sub-experiment refers to the sub-experiment shown in Figure 1. Parasite exposure refers to the miracidia of which parasite the snails were exposed. Days post exposure refers to the number of days between miracidia exposure and sample collection. Infection status refers to the infection status determined by the infection RD-PCR of Schols *et al.* [1] with reduced cycle numbers as mentioned in Hammoud *et al.* [2] and the metabarcoding infection output from Hammoud *et al.* [2]. Sample count lists the number of snail samples that meet each of the previously listed conditions and for which the bacterial communities were characterized using 16S rRNA gene metabarcoding. Counts marked with an asterisk were included in the first Illumina MiSeq run, samples with a ‘°’ were included in the second Illumina MiSeq run.

| Sub-experiment | Parasite exposure | Days post exposure | Infection status | Sample count |
| --- | --- | --- | --- | --- |
| Temporal signal mono-miracidial infection - Figure 1.B | *S. mansoni* Bre | 2 | Infected | 0 |
|  |  |  | Uninfected | 5* |
|  |  | 6 | Infected | 4* |
|  |  |  | Uninfected | 5* |
|  |  | 10 | Infected | 5* |
|  |  |  | Uninfected | 5* |
|  |  | 16 | Infected | 5* |
|  |  |  | Uninfected | 5* |
|  |  | 20 | Infected | 5* |
|  |  |  | Uninfected | 5* |
|  |  | 30 | Infected | 5* |
|  |  |  | Uninfected | 5* |
|  |  | 40 | Infected | 2* |
|  |  |  | Uninfected | 5* |
| 10-miracidial infections - Figure 1.C | *S. mansoni* Bre | 40 | Infected | 5*+19° |
|  |  |  | Uninfected | 2* |
|  | *S. mansoni* LE | 40 | Infected | 5*+19° |
|  |  |  | Uninfected | 2* |
|  | *S. rodhaini* | 40 | Infected | 5*+18° |
|  |  |  | Uninfected | 4* |
| Co-infection 10 miracidia each S*. mansoni* populations – Figure 1.D | *S. mansoni* Bre + *S. mansoni* LE | 2 | *S. mansoni* Bre | 0 |
|  |  |  | *S. mansoni* LE | 0 |
|  |  |  | *S. mansoni* Bre + *S. mansoni* LE | 0 |
|  |  |  | uninfected | 5* |
|  |  | 10 | *S. mansoni* Bre | 5* |
|  |  |  | *S. mansoni* LE | 5* |
|  |  |  | *S. mansoni* Bre + *S. mansoni* LE | 5* |
|  |  |  | uninfected | 1* |
|  |  | 40 | *S. mansoni* Bre | 4* |
|  |  |  | *S. mansoni* LE | 5* |
|  |  |  | *S. mansoni* Bre + *S. mansoni* LE | 5* |
|  |  |  | uninfected | 1* |
| Co-infection 10 miracidia each S*. mansoni* and *S*. *rodhaini* – Figure 1.E | *S. mansoni* LE + *S. rodhaini* | 2 | *S. rodhaini* | 3* |
|  |  |  | *S. mansoni* LE | 0 |
|  |  |  | *S. rodhaini + S. mansoni* LE | 0 |
|  |  |  | uninfected | 5* |
|  |  | 10 | *S. rodhaini* | 0 |
|  |  |  | *S. mansoni* LE | 5* |
|  |  |  | *S. rodhaini + S. mansoni* LE | 3* |
|  |  |  | uninfected | 0 |
|  |  | 40 | *S. rodhaini* | 1* |
|  |  |  | *S. mansoni* LE | 5* |
|  |  |  | *S. rodhaini + S. mansoni* LE | 2* |
|  |  |  | uninfected | 2* |
| Snails never exposed to miracidia – Naïve snails | NA | 0 | NA | 5* |
|  | NA | 40 | NA | 5* |

| Exposure | *Schistosoma* *mansoni* BRE + *Schistosoma* *mansoni* LE | | | | | | | | | | | |  | |
| --- | --- | --- | --- | --- | --- | --- | --- | --- | --- | --- | --- | --- | --- | --- |
| Days | 2 (n = 32) | | | | 10 (n = 32) | | | | 40 (n = 27) | | | |  |  |
| Strain present | None | Only SmBRE | Only SmLE | Both | None | Only SmBRE | Only SmLE | Both | None | Only SmBRE | Only SmLE | Both |  |  |
| Observed | 32 | 0 | 0 | 0 | 3 | 5 | 6 | 19 | 1 | 4 | 16 | 6 |  |  |
|  | | | | | | | | | | | | | | |
| Exposure | *Schistosoma* *mansoni* LE + *Schistosoma* *rodhaini* | | | | | | | | | | | |  | |
| Days | 2 (n = 32) | | | | 10 (n = 31) | | | | 40 (n = 30) | | | |  |  |
| Strain present | None | Only SmLE | Only Sr | Both | None | Only SmLE | Only Sr | Both | None | Only SmLE | Only Sr | Both |  |  |
| Observed | 29 | 0 | 3 | 0 | 0 | 28 | 0 | 3 | 1 | 26 | 0 | 3 |  |  |
|  | | | | | | | | | | | | | | |
| Exposure | *Schistosoma* *mansoni* BRE | | | | | | | | | | | | | |
| Days | 2 (n =20) | | 6 (n =20) | | 10 (n =20) | | 16 (n =20) | | 20 (n =20) | | 30 (n =20) | | 40 (n =20) | |
| Strain present | None | SmBRE | None | SmBRE | None | SmBRE | None | SmBRE | None | SmBRE | None | SmBRE | None | SmBRE |
| Observed | 20 | 0 | 16 | 4 | 15 | 5 | 11 | 9 | 12 | 8 | 11 | 9 | 20 | 2 |
|  | | | | | | | | | | | | | | |
| Exposure | SmBRE | | SmLE | | Sr | | unexposed | |  | | | | | |
| Days | 40 (n =27) | | 40 (n =26) | | 40 (n =28) | | 0 (n =5) | 40 (n =5) |  |  |  |  |  |  |
| Strain present | None | SmBRE | None | SmLE | None | Sr | None | None |  |  |  |  |  |  |
| Observed | 1 | 26 | 2 | 24 | 5 | 23 | 5 | 5 |  |  |  |  |  |  |

Suppl. Table 2: Rates of parasite infection in function of the exposure condition, days post exposure based on the original dataset of Hammoud *et al.* [2] from which the samples of Suppl. Table 1 were selected.


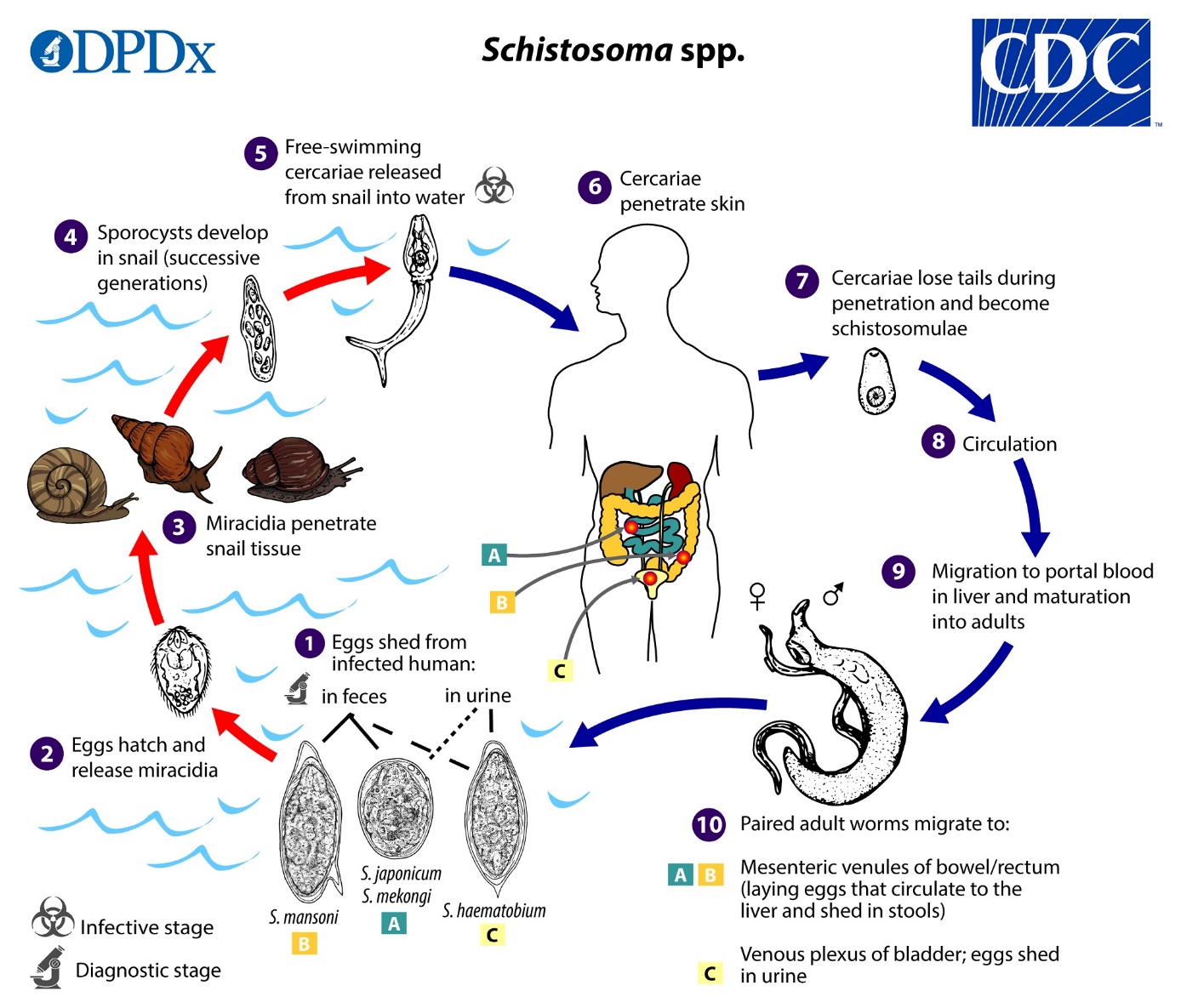


Suppl. Figure 1: Life cycle of *Schistosoma mansoni* (B), highlighting its key developmental stages. Eggs are excreted in feces (1) and, under suitable environmental conditions, hatch into free-swimming miracidia (2) that actively seek and infect specific freshwater snails as intermediate hosts (3). Within the snail, the parasite undergoes two sporocyst generations (4), culminating in the production of cercariae (5). These cercariae emerge from the snail, swim freely in water, and penetrate the skin of a human host (6). After entry, they lose their tails and become schistosomulae (7), which migrate (8) through the bloodstream, pass through the lungs and heart, and mature in the liver (9). Adult male and female worms pair and settle primarily in the mesenteric veins draining the large intestine (10). Once established, the females lay eggs that traverse the intestinal wall and are ultimately expelled in feces (1). The diagram also depicts other *Schistosoma* species for comparison (*Schistosoma japonicum* and *Schistosoma mekongi*, A; *Schistosoma haematobium*, C). Source: CDC (https://www.cdc.gov/dpdx/schistosomiasis/index.html, accessed on 04 Aug. 2025). Disclaimer: Reference to specific commercial products, manufacturers, companies, or trademarks does not constitute its endorsement or recommendation by the U.S. Government, Department of Health and Human Services, or Centers for Disease Control and Prevention.

(Figure continues on the next page)


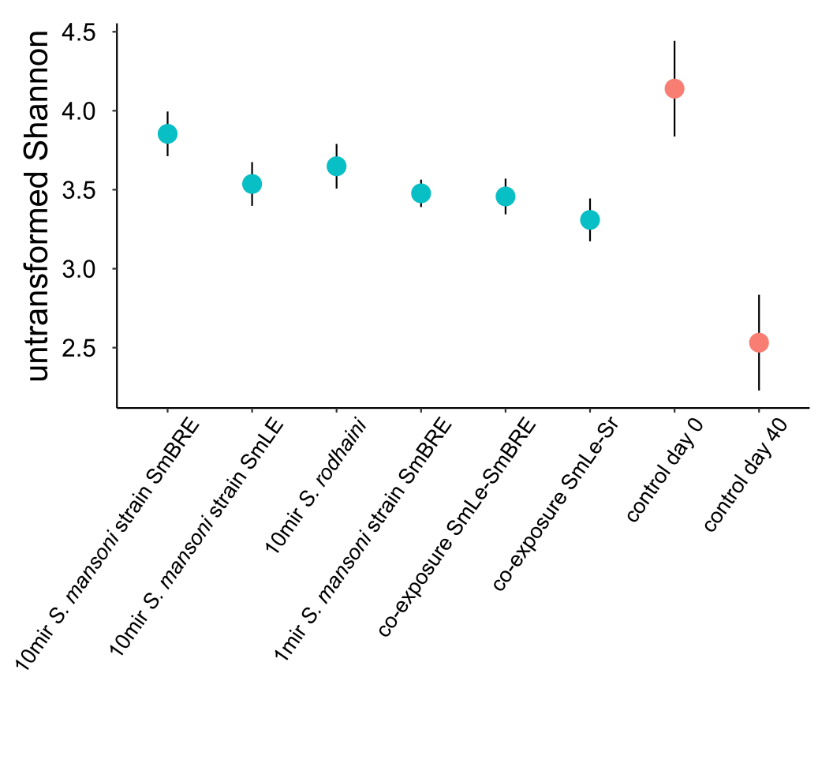

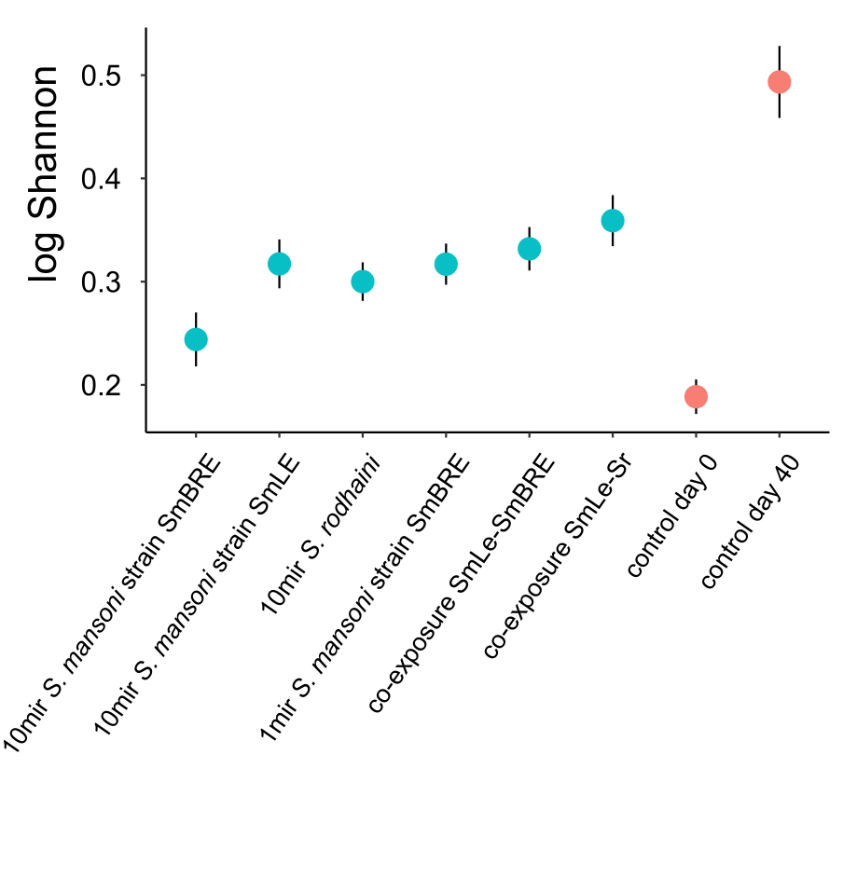

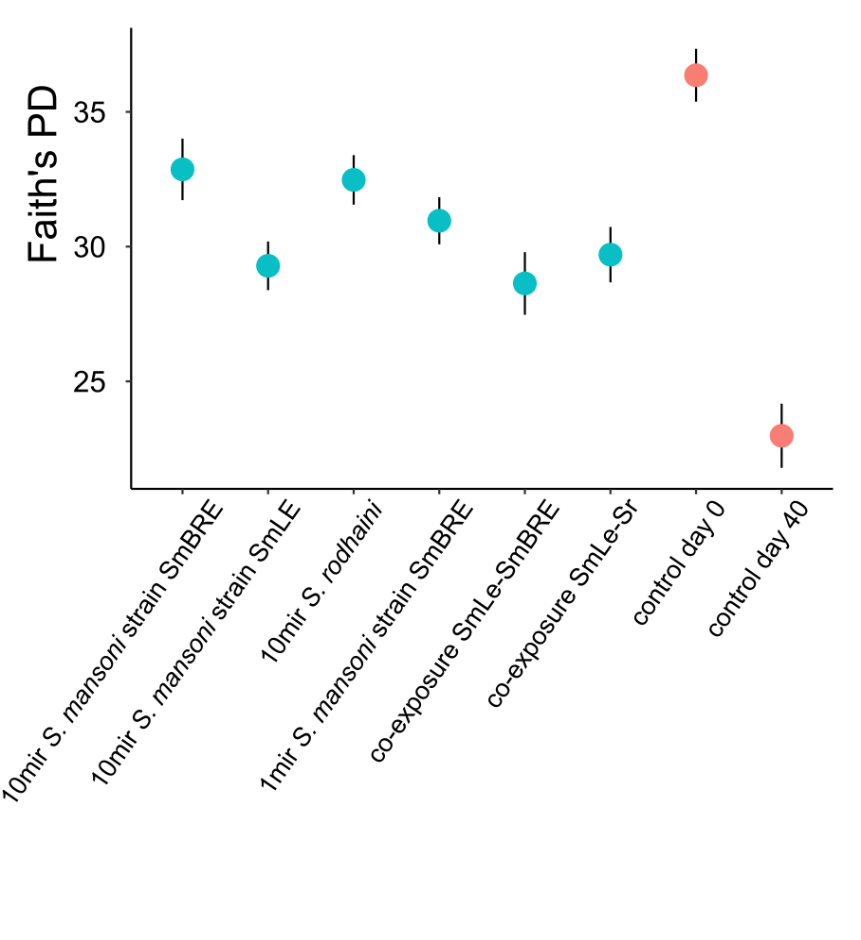


**A)**

**B)**

**C)**


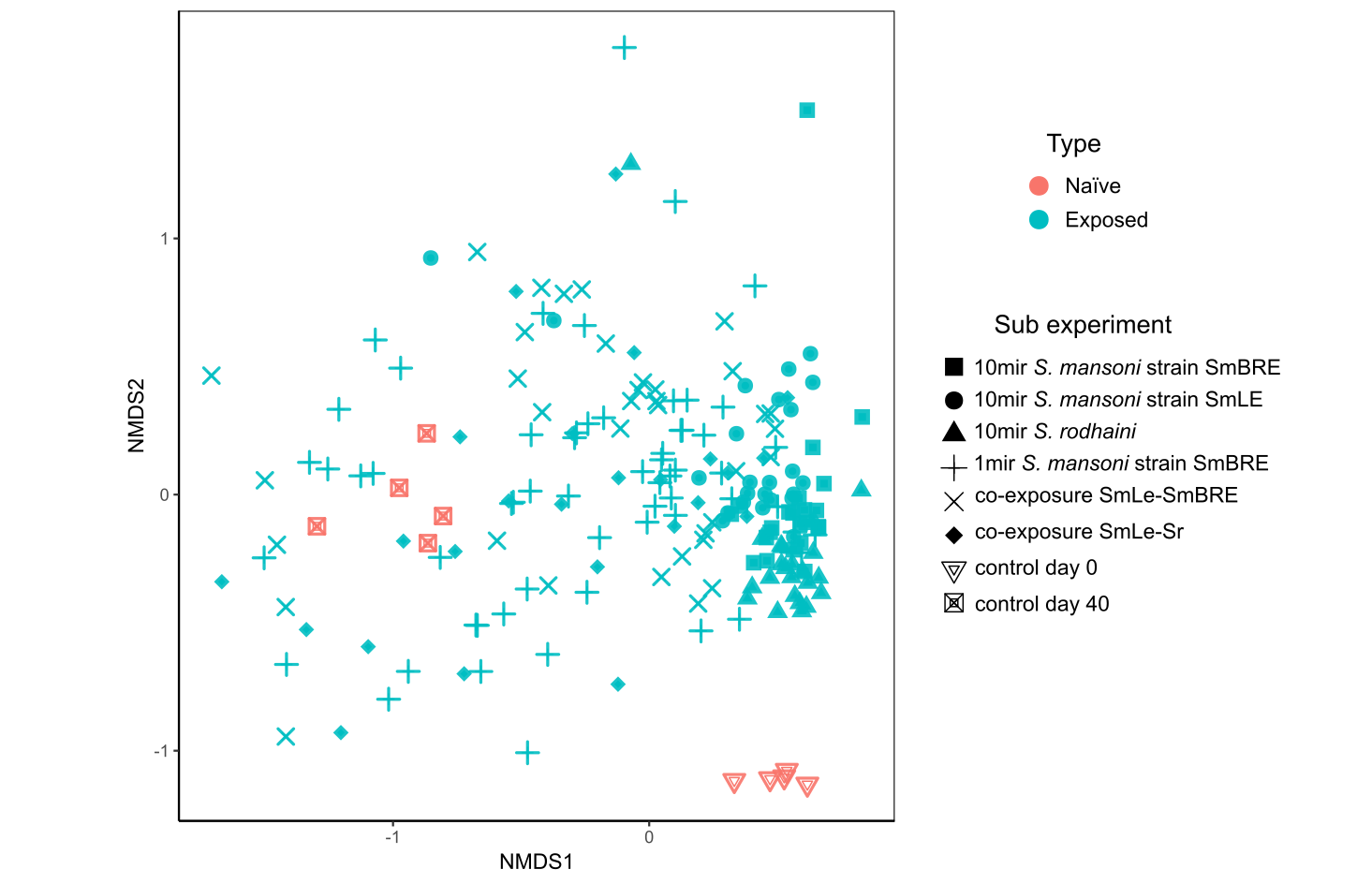

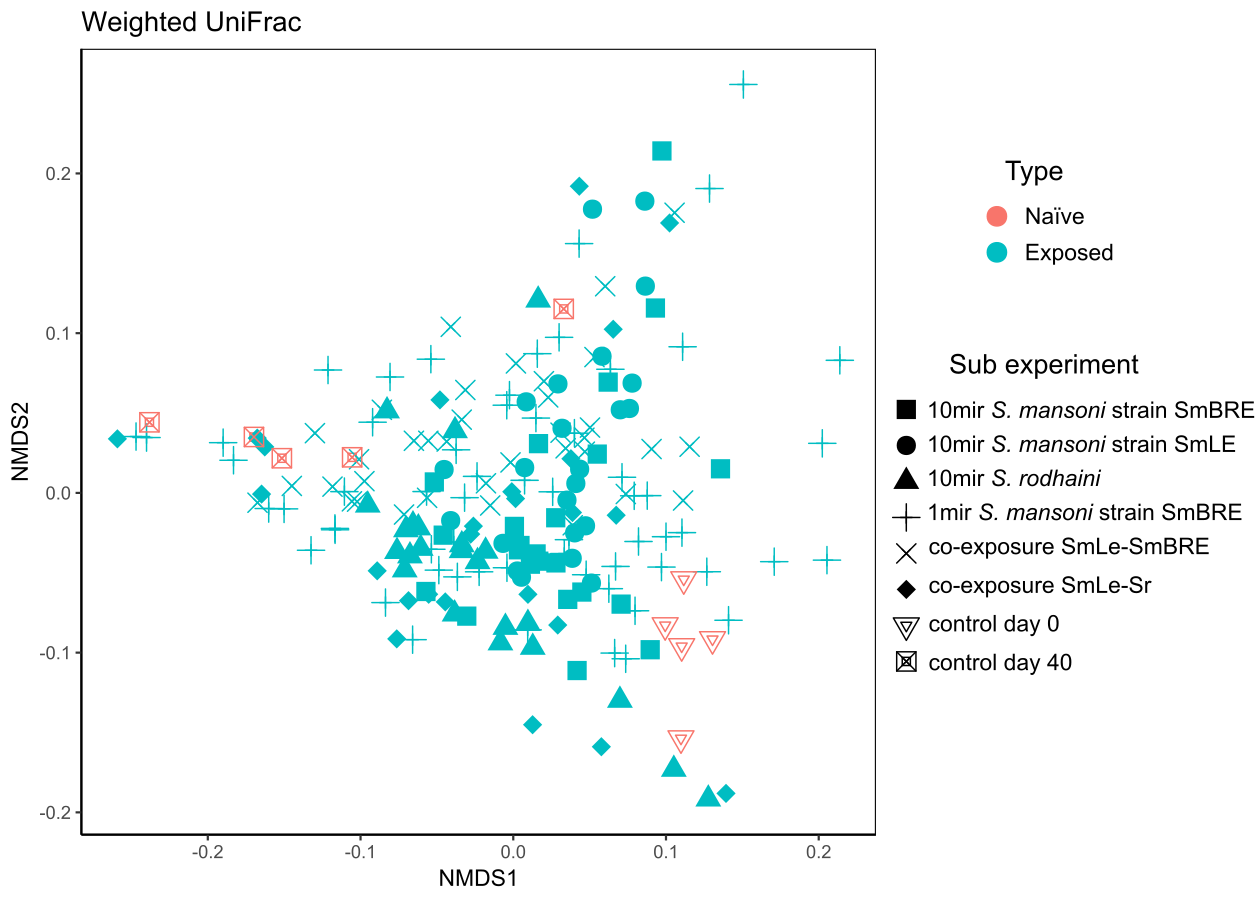


**D)**

**E)**

(Figure continues on the next page)

Suppl. Figure 2: Comparing miracidia exposed snail samples to naïve (control) snails across all sub-experiments. A) Untransformed Shannon diversity, for illustration. B) log-transformed Shannon diversity metric comparing the various sub experiments. C) Faith’s Phylogenetic diversity comparing the various sub experiments. D) NMDS analysis of the Bray-Curtis dissimilarity measure across the entire dataset. E) NMDS analysis of the weighted Unifrac distance measure across the entire dataset, which did not converge. F) NMDS analysis of the unweighted Unifrac distance measure across the entire dataset. Color indicates parasite exposed (blue) and unexposed or naïve (pink) snails. Sub-experiment is indicated by different shapes. For significance information see main text.


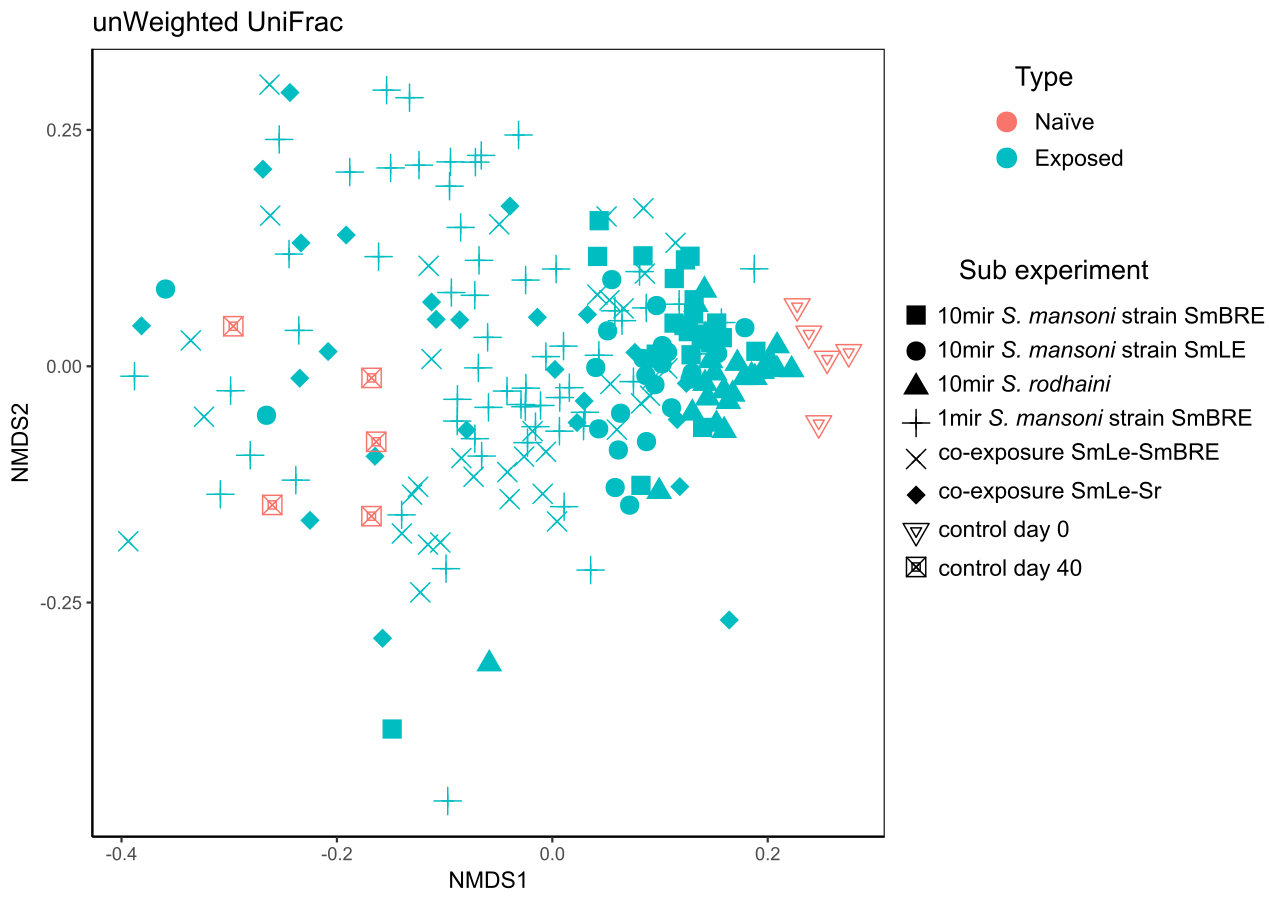


**F)**


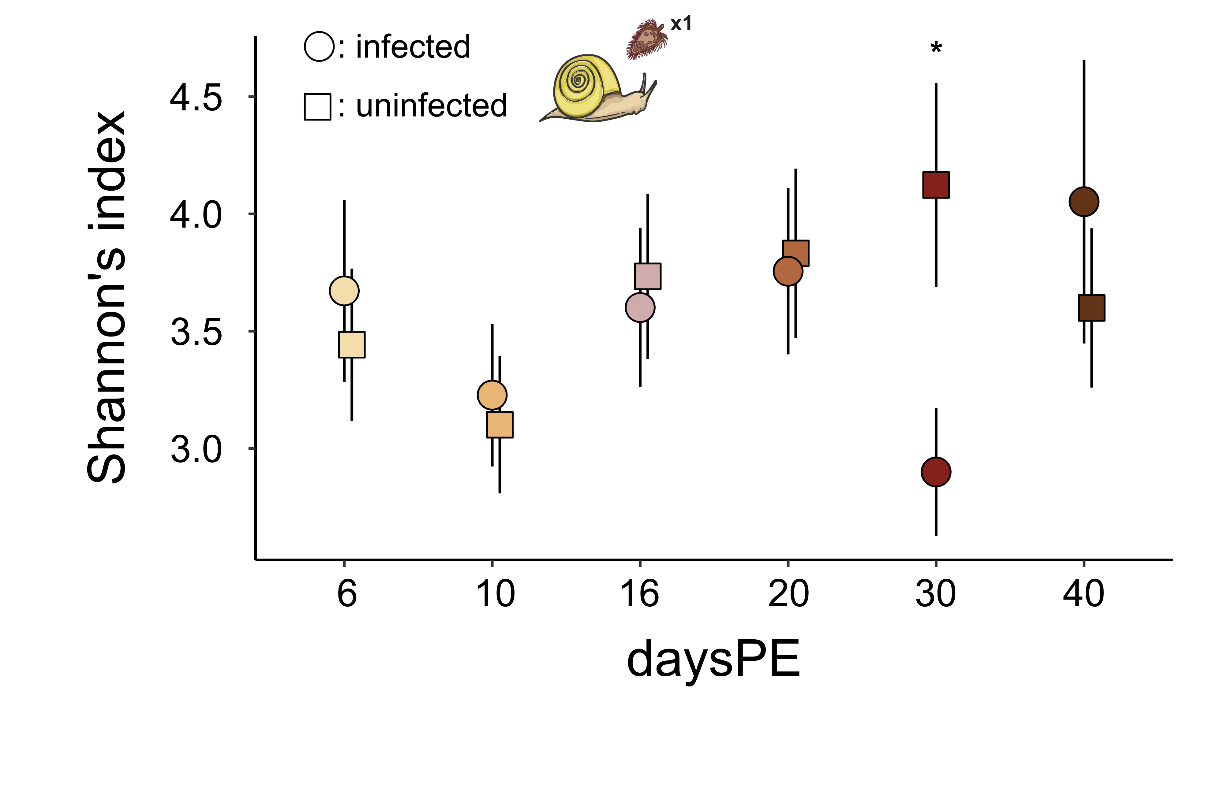


Suppl. Figure 3: The temporal pattern throughout schistosome infection development. Shannon diversity metric for infected vs uninfected samples across various days post miracidium exposure (daysPE). Circles indicate infected samples, squares uninfected samples. The asterisk indicates the significant pairwise difference between infected and uninfected samples at 30 daysPE (p<0.05). A single outlier with Bonferroni p-value <0.05 has been removed from this dataset (uninfected snail at 30 daysPE).

Suppl. Figure 4: The Shannon diversity related to the proportion of reads attributed to SmBRE as determined by the HTAS protocol of Hammoud *et al.*, [2]. A) The Shannon diversity metric for different proportions of reads attributed to parasite infections per sample across various days post miracidium exposure (daysPE). B) The Faith’s PD metric for different proportions of reads attributed to parasite infections per sample across various days post miracidium exposure (daysPE). C) The Shannon diversity metric for different proportions of reads attributed to parasite infections per samples at 30 daysPE. D) The Shannon diversity metric for different proportions of reads attributed to parasite infections per samples at 30 daysPE. Panels C and D represent the only significant daysPE condition (30 daysPE) shown in panels A and B, respectively. A single outlier with Bonferroni p-value <0.05 has been removed from this dataset (uninfected snail at 30 daysPE).


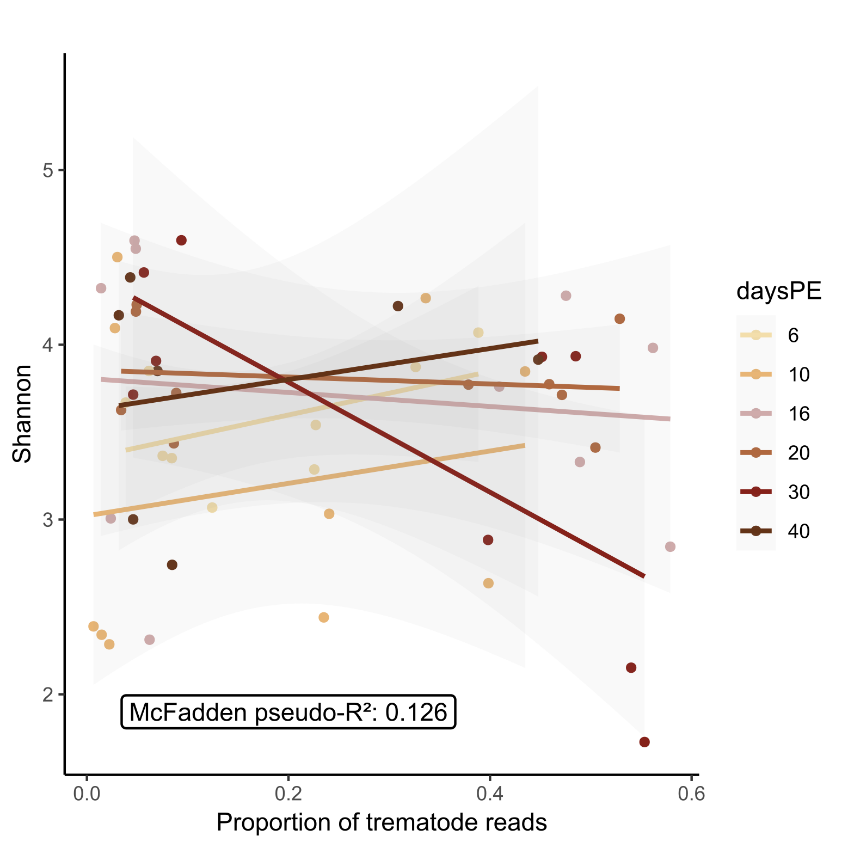

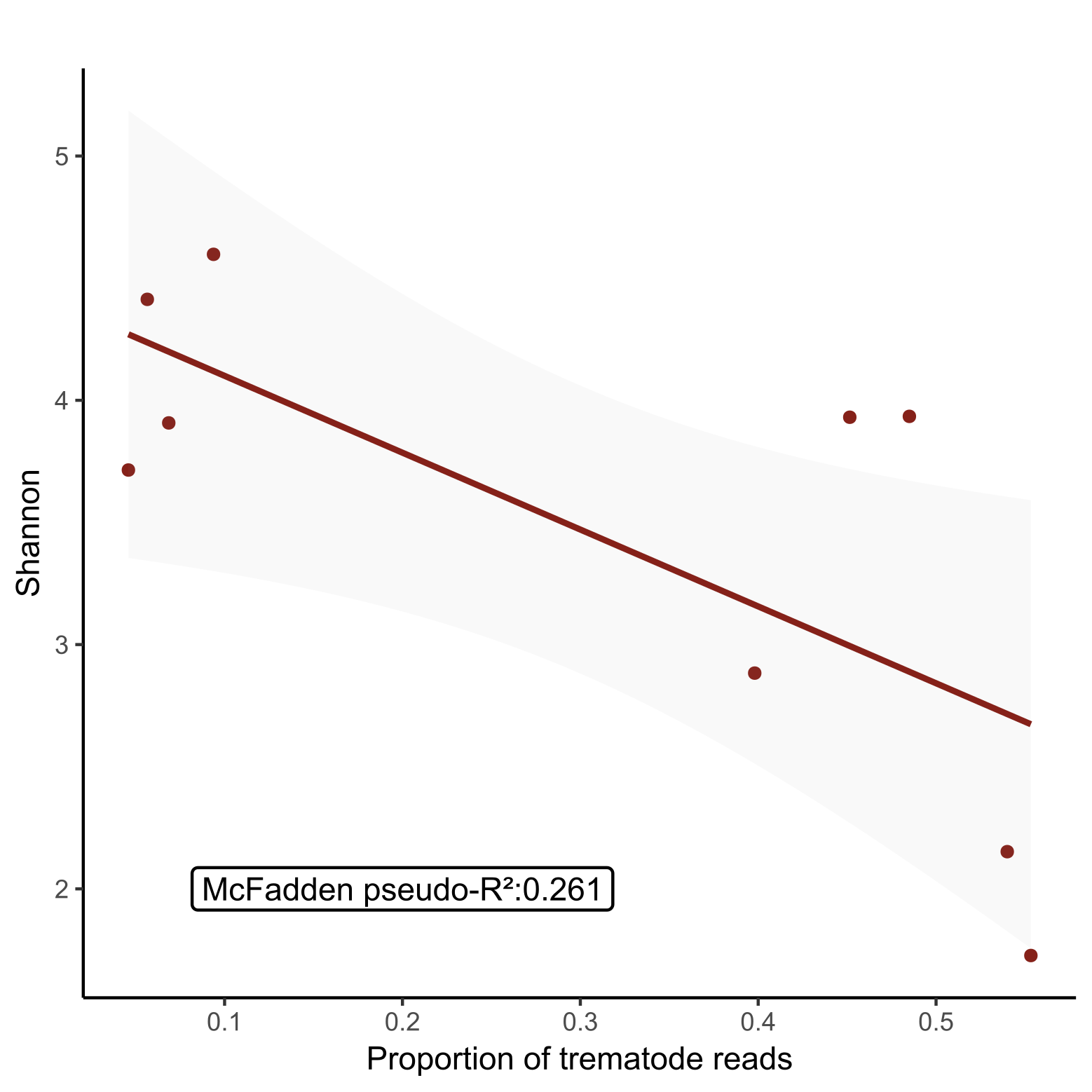


**A)**


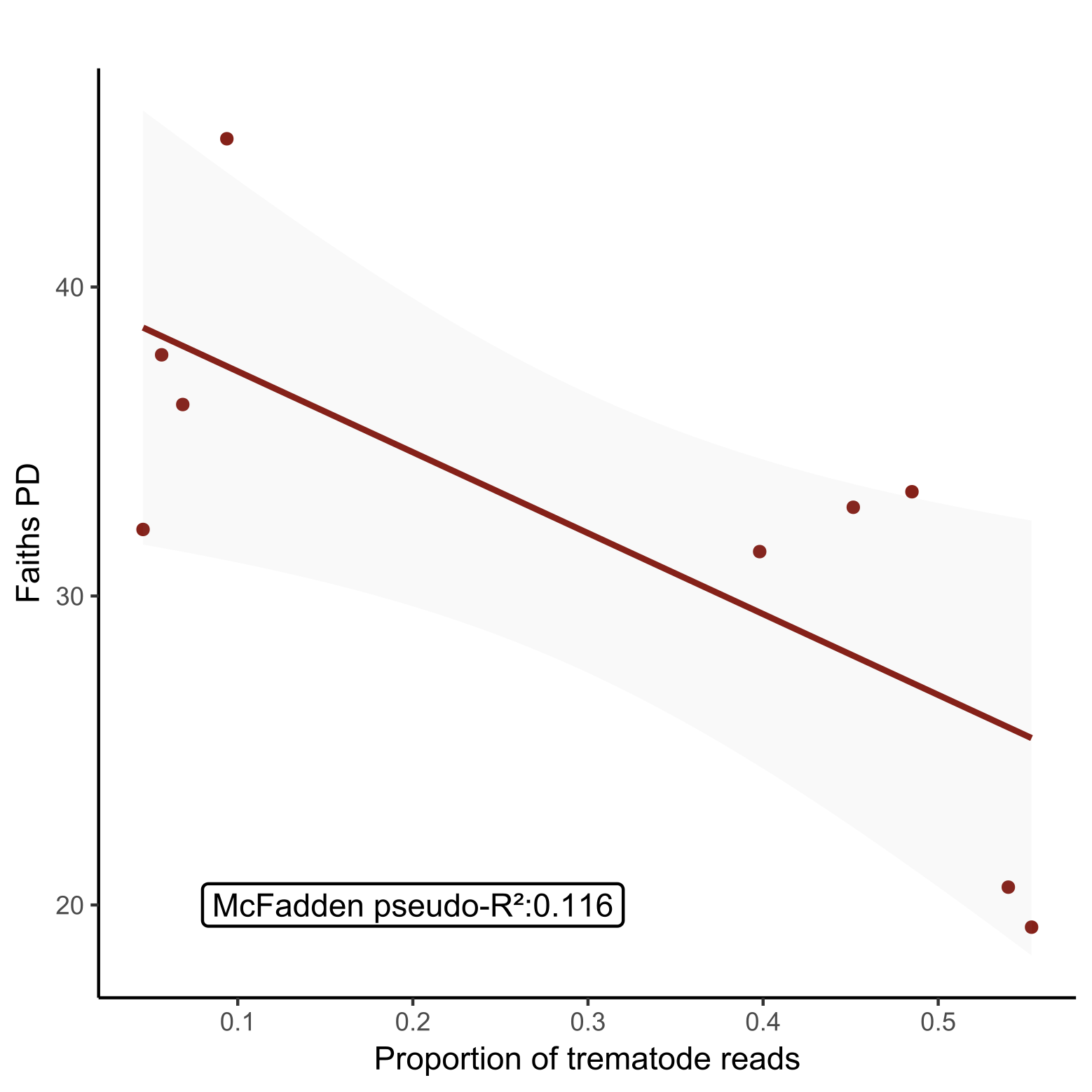

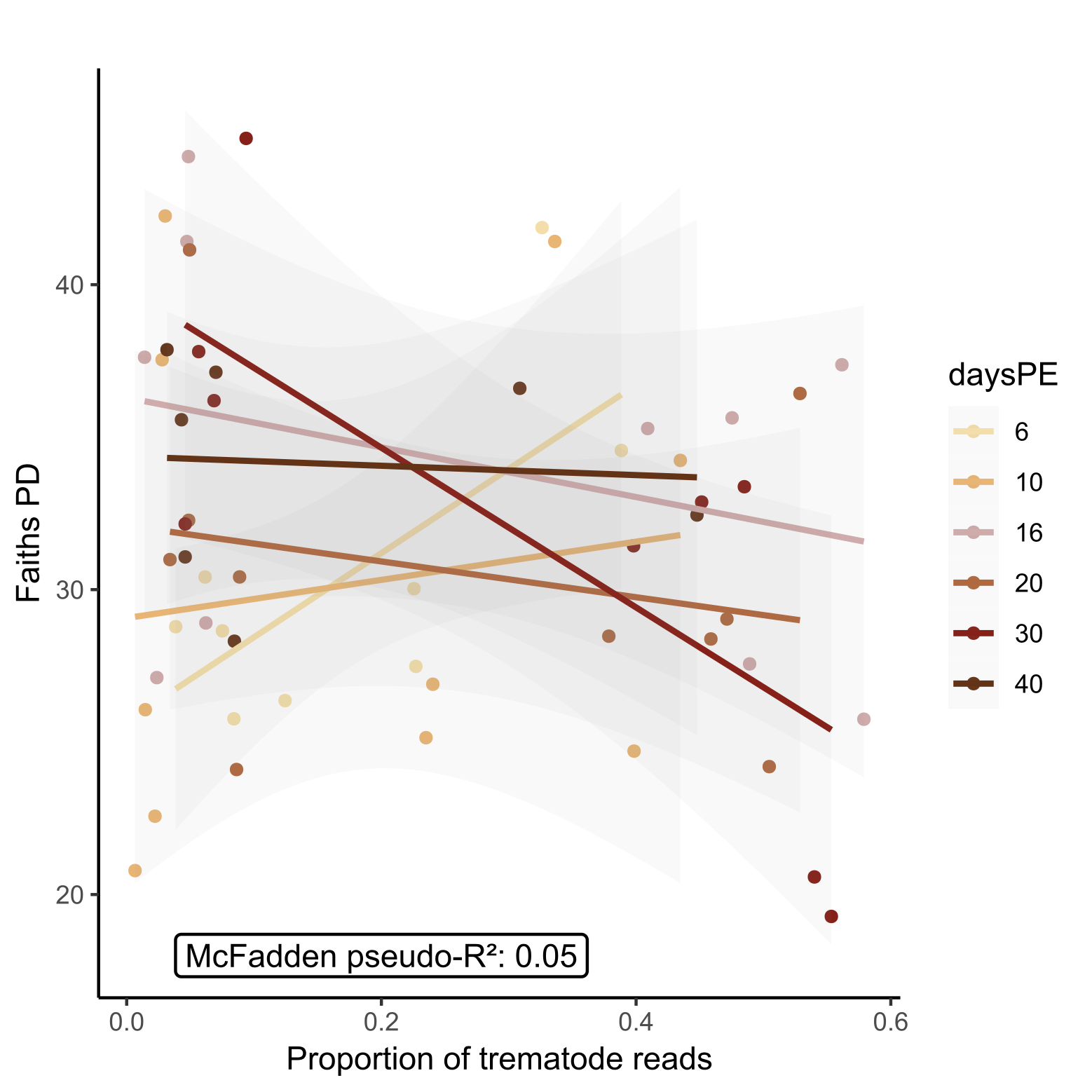


**B)**

**C)**

**D)**

Suppl. Figure 5: The effect of including the identified outlier with Bonferroni p-value <0.05 (uninfected snail at 30 daysPE) on the temporal pattern throughout schistosome infection development. A) Shannon diversity metric for infected vs uninfected samples across various days post miracidium exposure (daysPE). B) Faith’s Phylogenetic diversity metric for infected vs uninfected samples across various days post miracidium exposure (daysPE). Circles indicate infected samples, squares uninfected samples. C) The Shannon diversity metric for different proportions of reads attributed to parasite infections per samples at 30 daysPE. D) The Faiths PD metric for different proportions of reads attributed to parasite infections per samples at 30 daysPE. The outlier is indicated in red.


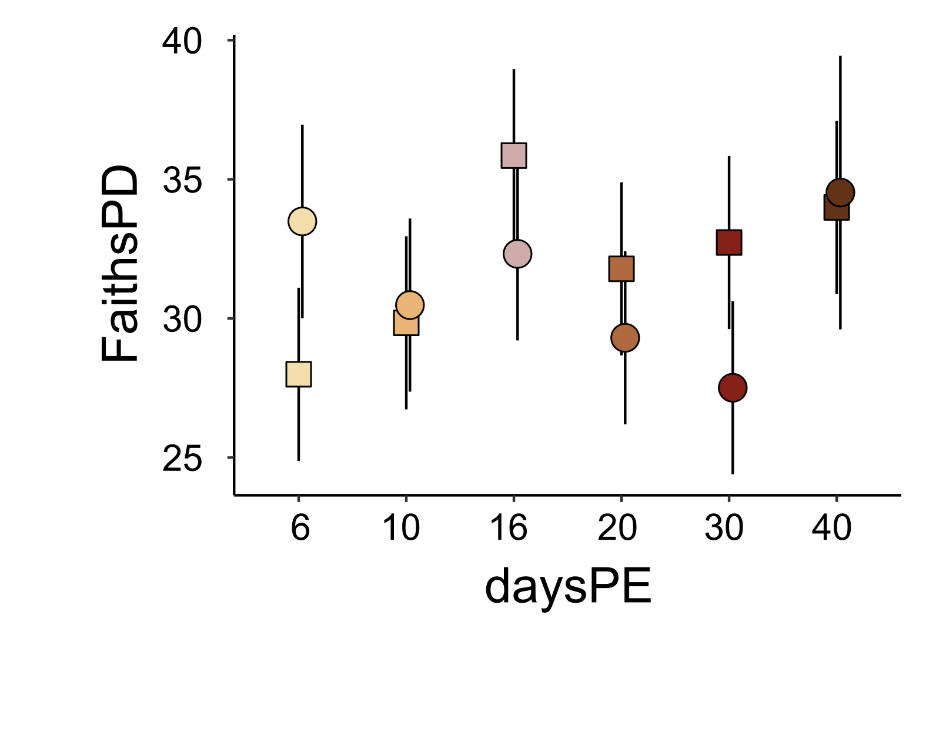

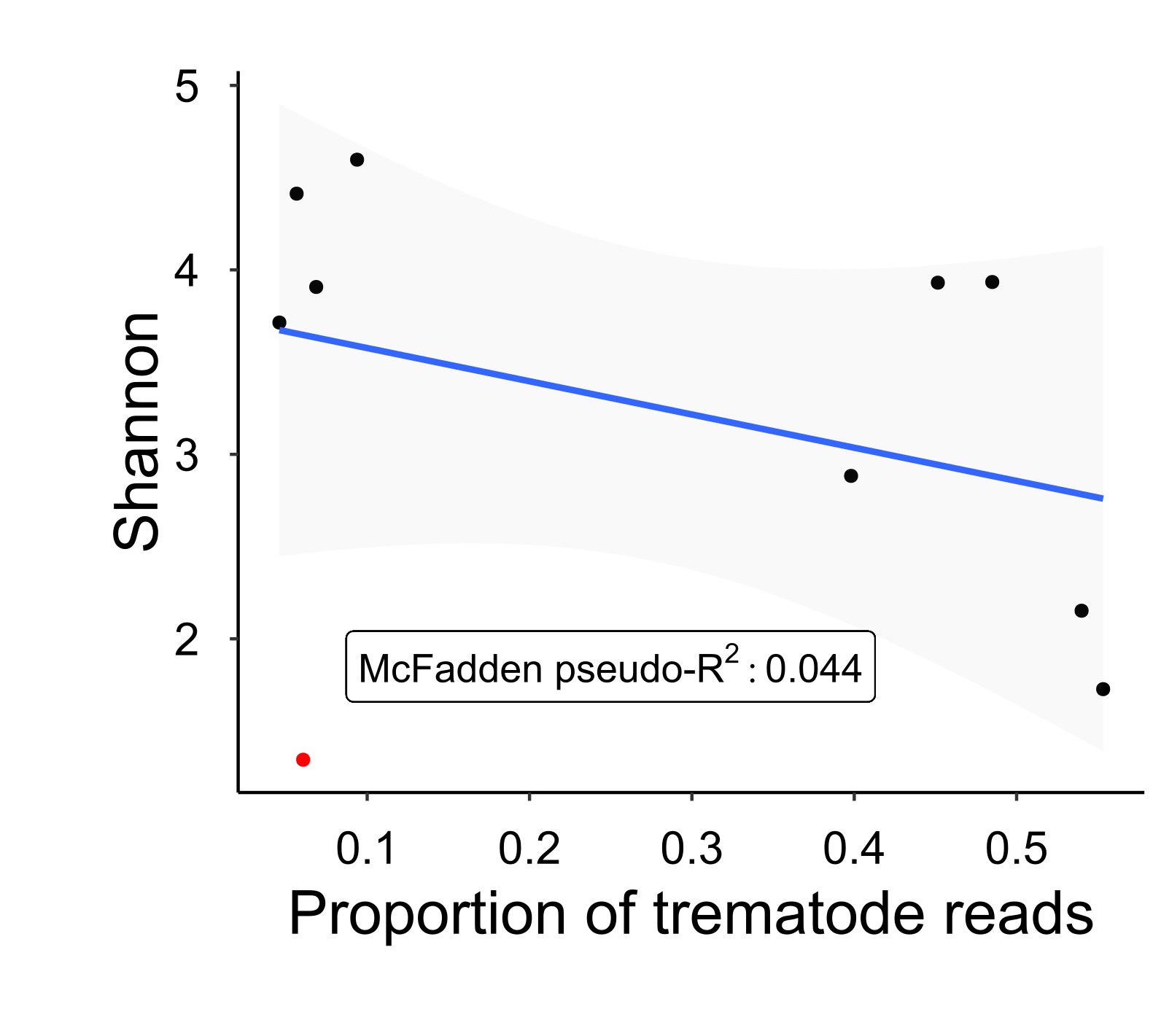


**B)**

**C)**


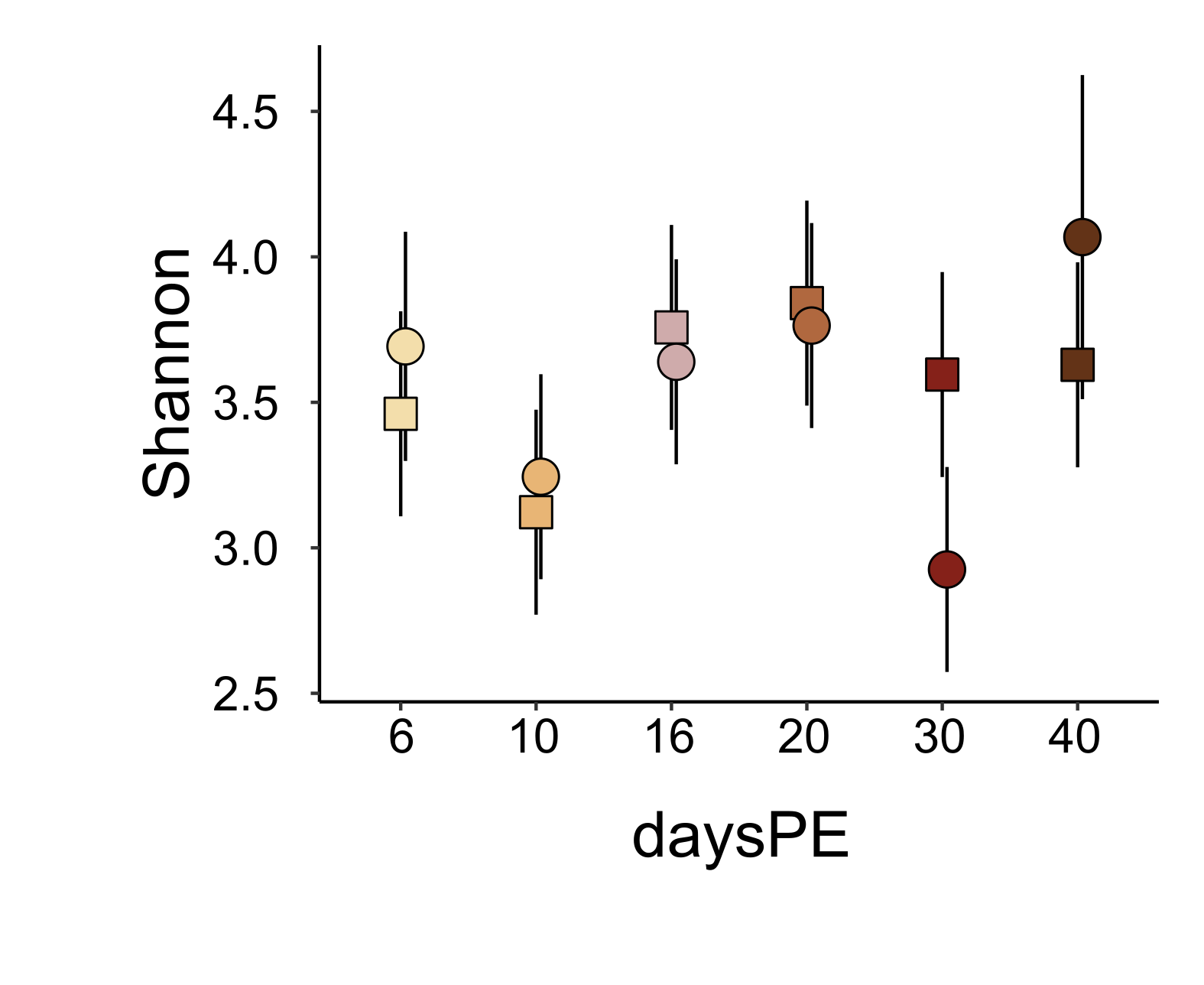


**A)**


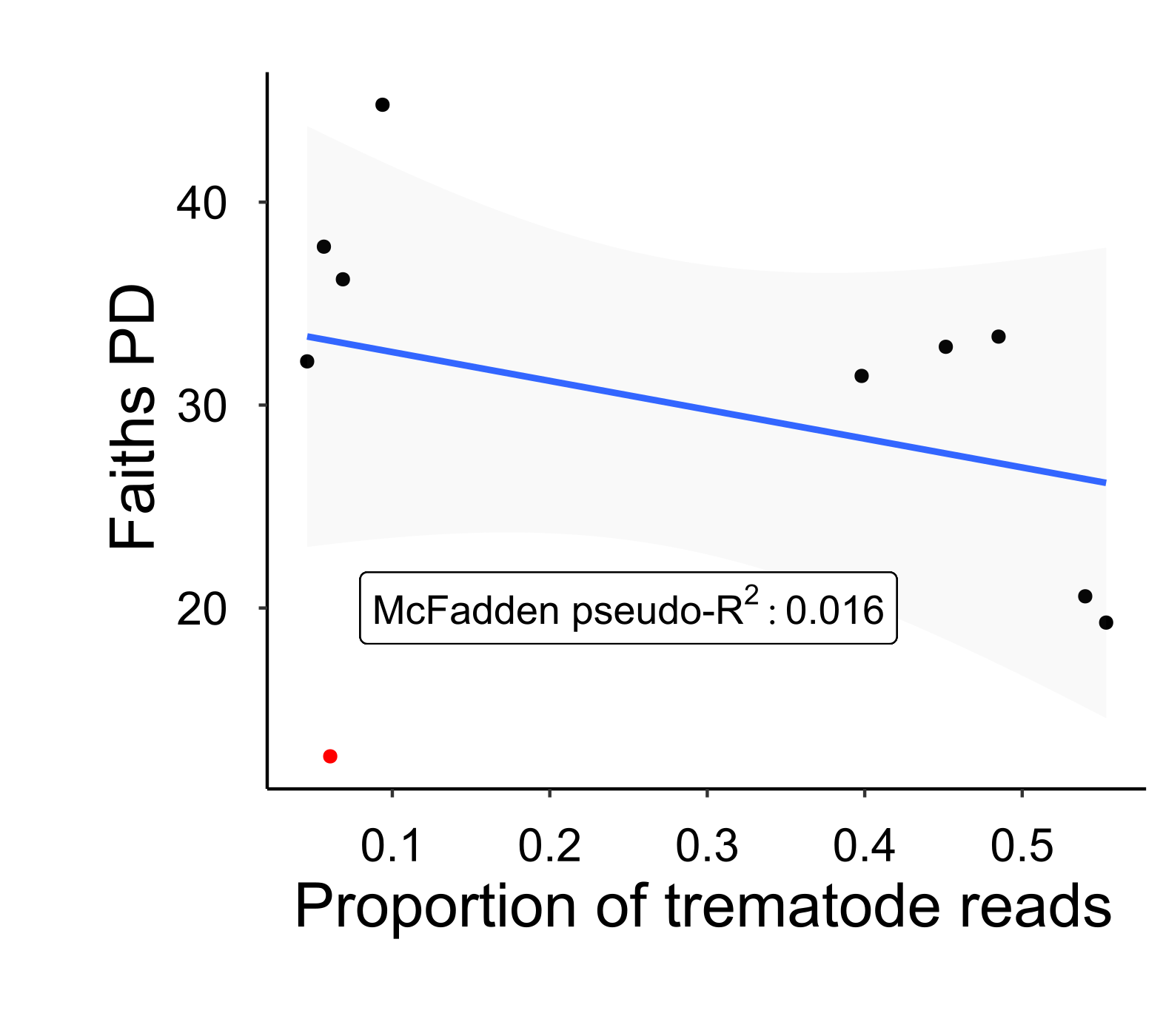


**D)**


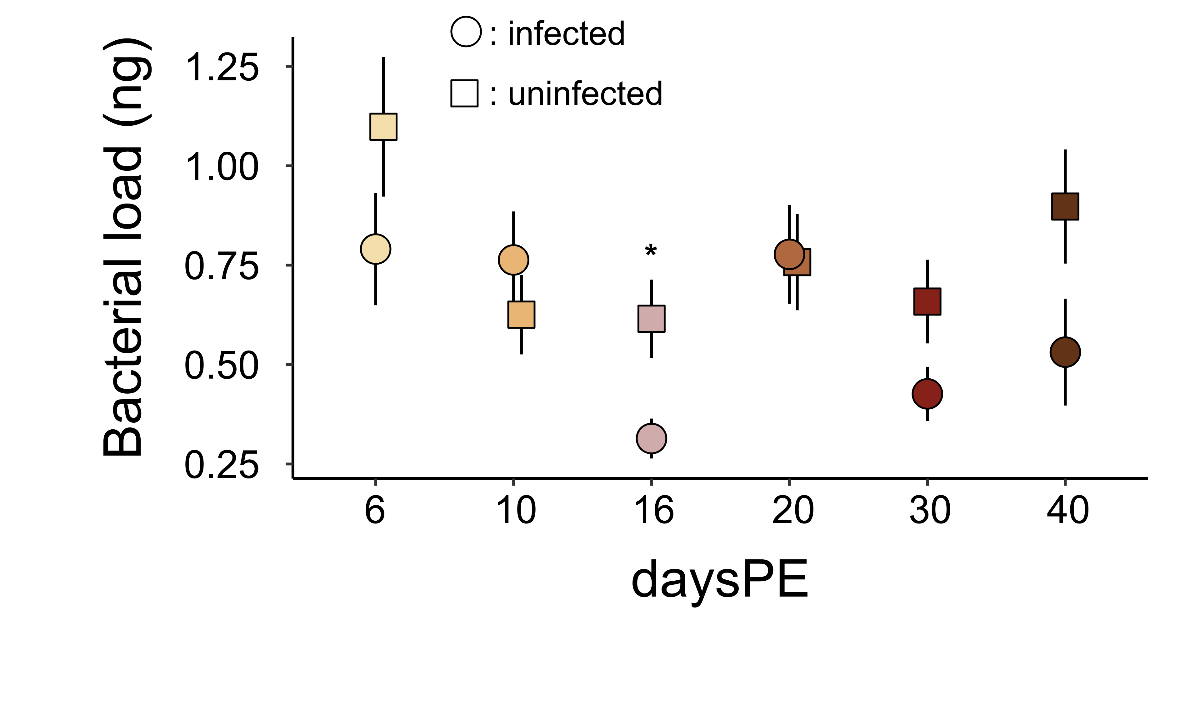


Suppl. Figure 6: Bacterial load (ng) as determined through qPCR measurements. This quantity of DNA was used as PCR-input from the 10 ng of total DNA used. Total DNA encompasses snail, parasite, bacterial, and other organisms' DNA. Circles indicate infected samples, squares uninfected samples. The asterisk indicates the significant pairwise differences (p<0.05). DaysPE = days post exposure. The difference in means between infected and uninfected samples is maginally non-signficant at 30 and 40 daysPE (p-value=0.06 and p-value=0.09, resp.).


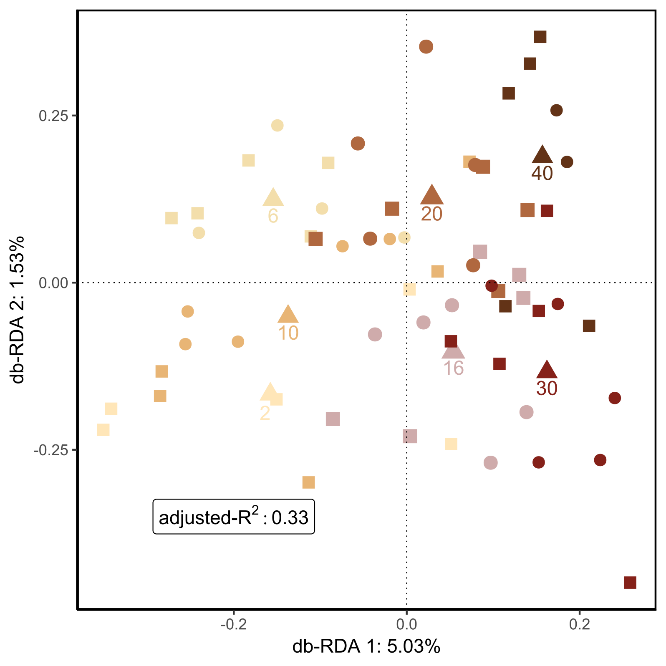

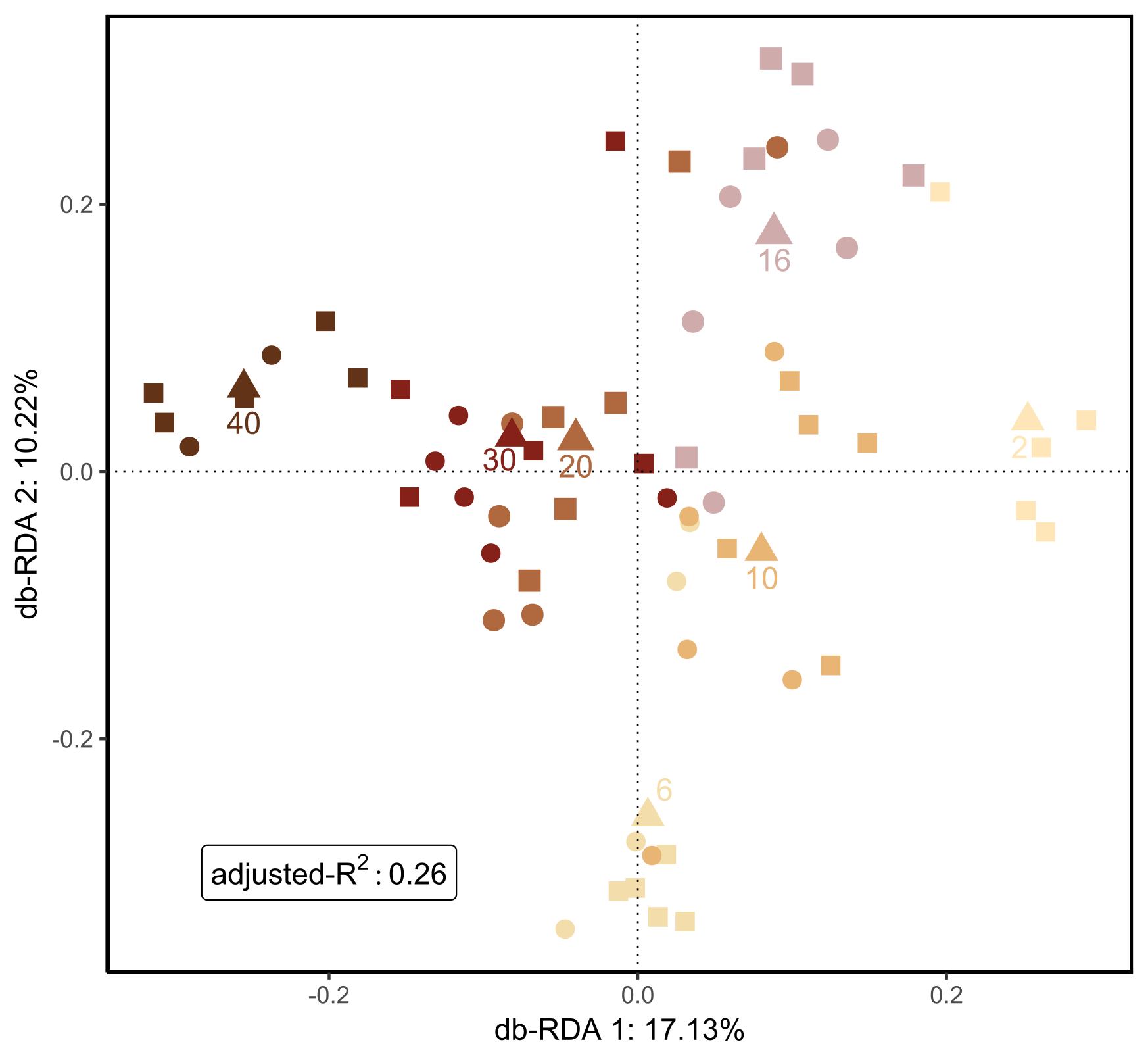


**A)**

**B)**

Suppl. Figure 7: The beta diversity of infected vs uninfected samples across various days post SmBRE miracidium exposure (daysPE). A) Weighted Unifrac distance as shown by RDA plot of the bacterial community adjusted for the different days post exposure. Ordistep model selection for RDA indicated that infection status did not affect the bacterial community (F=0.86, p-value=0.53), while daysPE did have a significant effect (F=5.15, p-value < 0.001). B) Unweighted Unifrac distance as shown by RDA plot of the bacterial community adjusted for the different days post exposure. Ordistep model selection for RDA indicated that infection status did not affect the bacterial community (F=0.85, p-value=0.66), while daysPE did have a significant effect (F=4.46, p-value < 0.001). Infection status was selected out of the RDA by ordistep but is indicated for illustration purposes. Circles indicate infected samples, squares uninfected samples. The triangles are the centroids of all samples belonging to the specific post exposure day. All samples at 2 daysPE are classified as uninfected since the infection status cannot be reliably determined this soon after exposure.


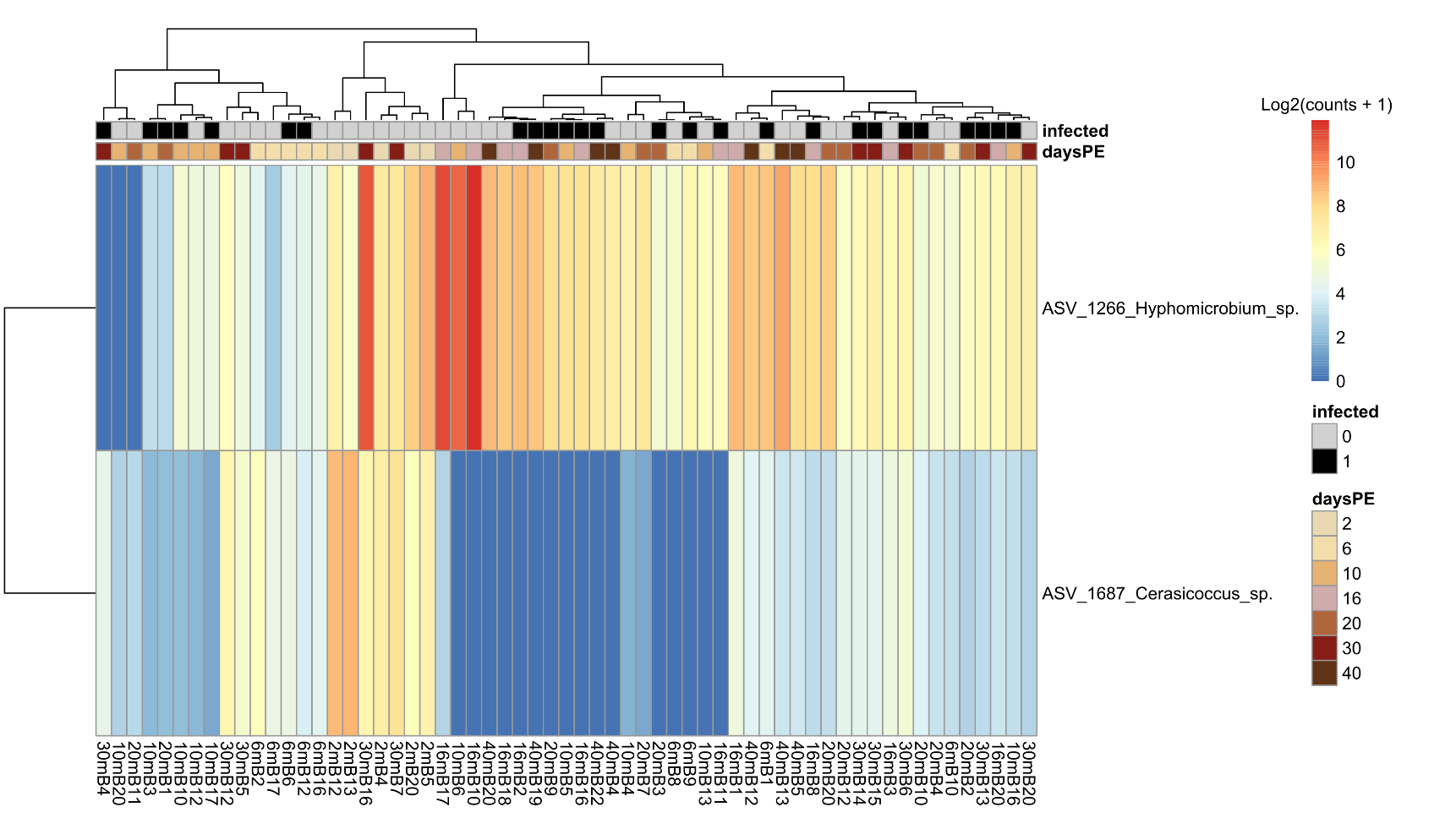


Suppl. Figure 8: A heatmap showing the Log2(read counts+1) for each sample at the genus level as selected for through DeSeq2 for significantly changing read counts with adjusted p-values <0.05. Clustering on top is based on sample similarity based on significantly different ASVs at genus level.


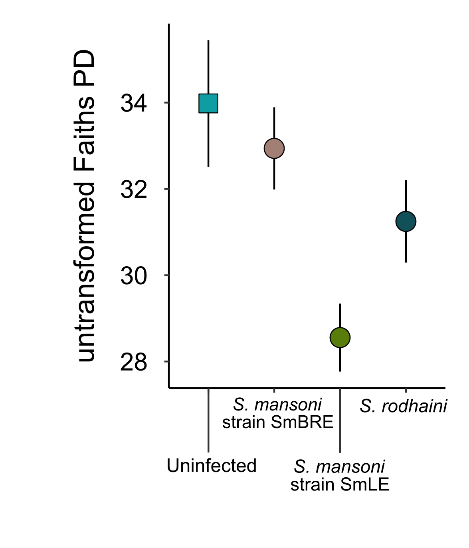

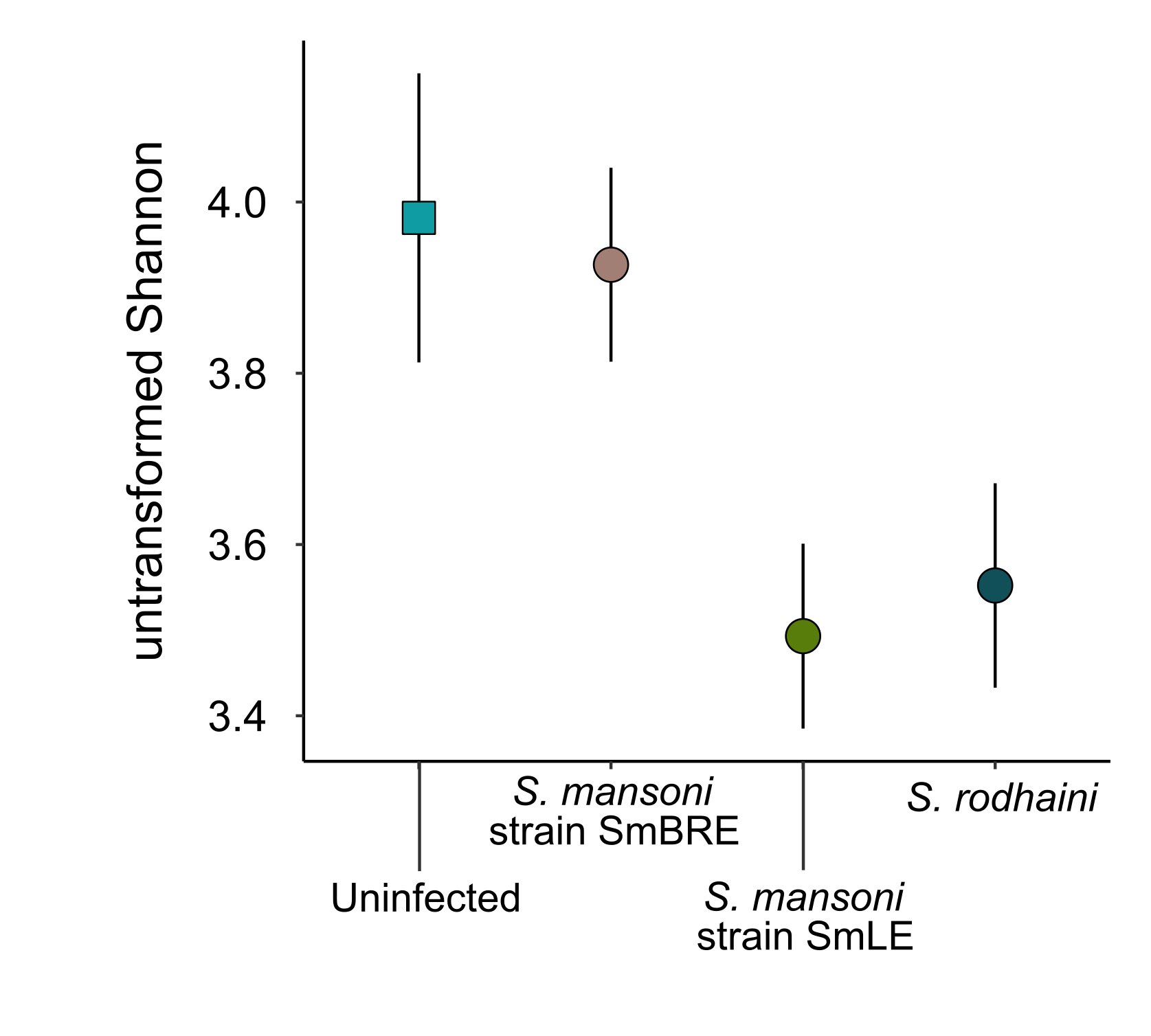


**A)**

**B)**

**C)**


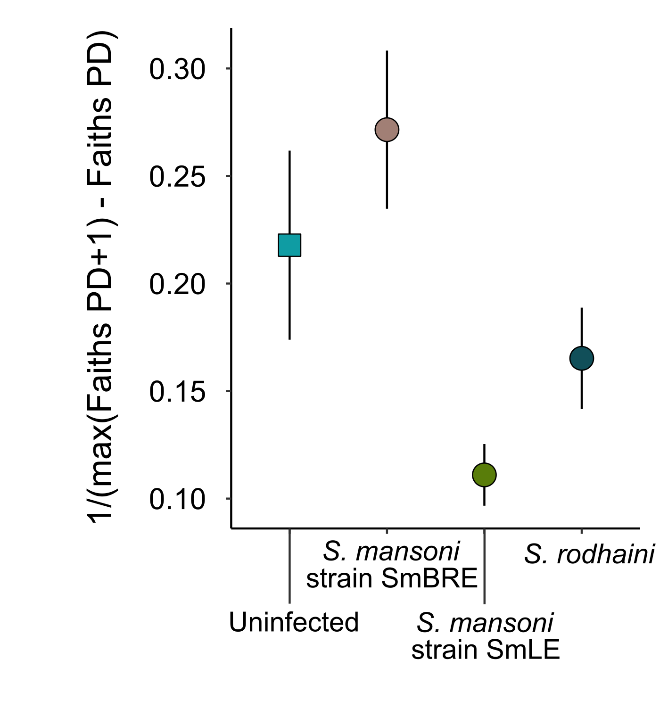


a

b

ab

a

Suppl. Figure 9: A) Untransformed Shannon diversity. B) Untransformed Faiths phylogenetic diversity. Uninfected samples from all exposure experiments are combined under “Uninfected” as selected for by the model. Error bars indicate a single standard deviation. C) Inverse transformed Faiths phylogenetic diversity metric (alpha diversity), based on mean values with error bars representing a single standard deviation. Letters indicate significant differences between means.


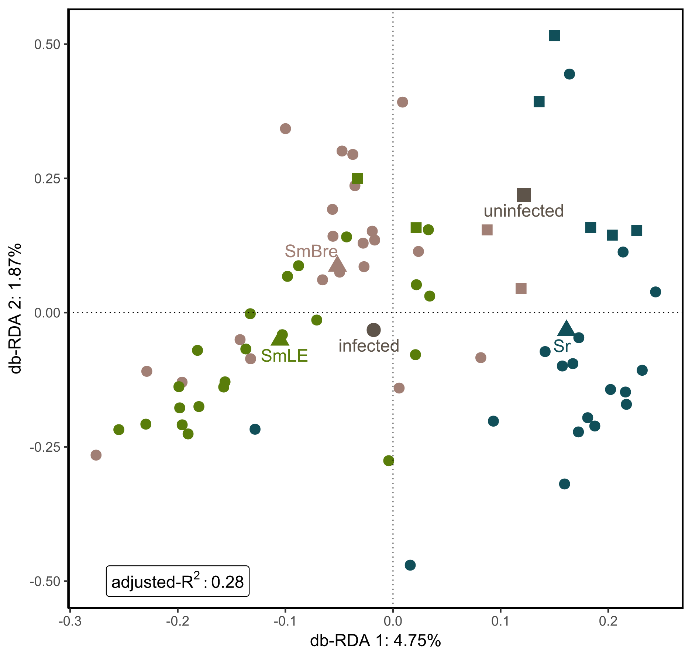

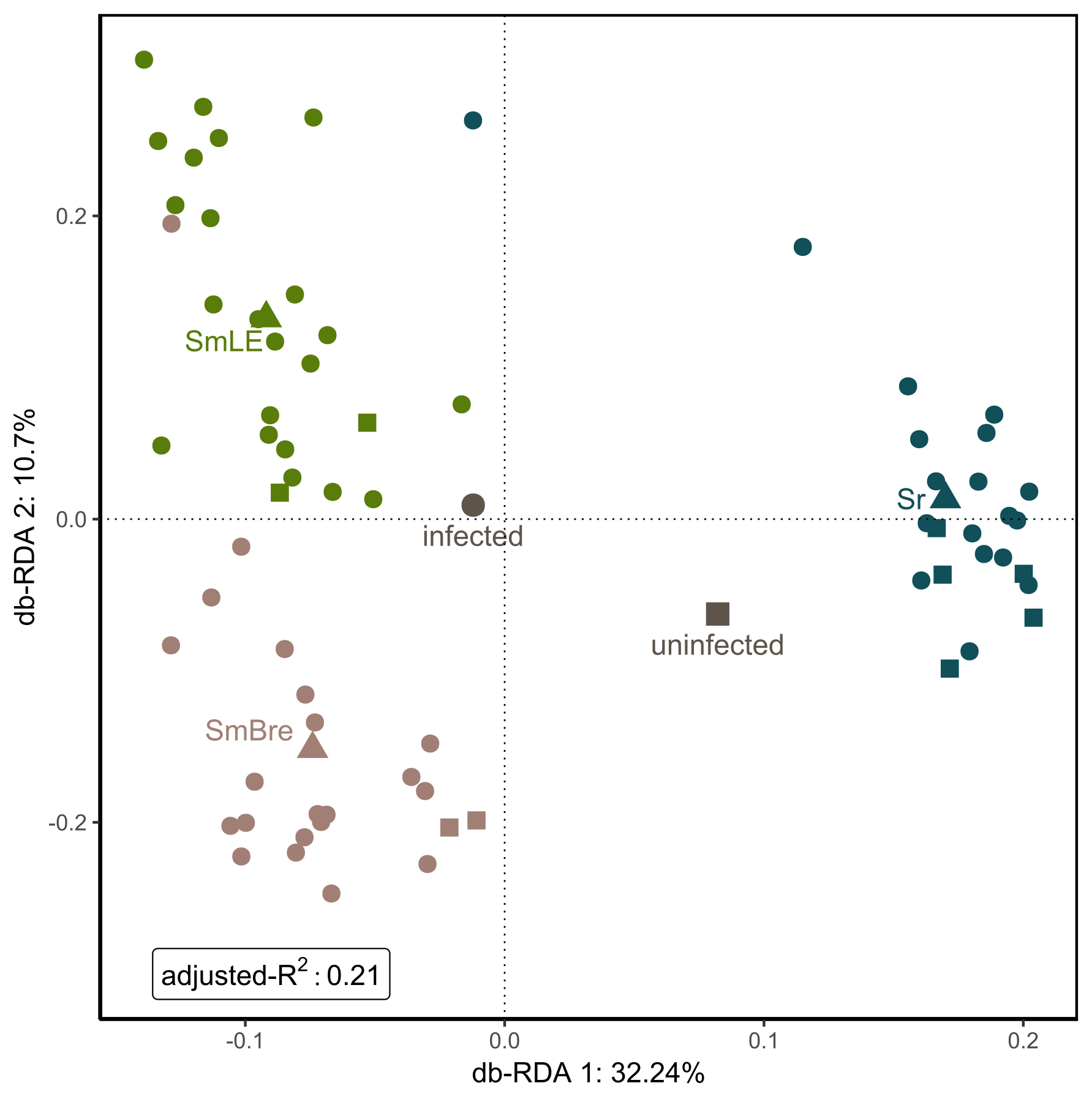


**A)**

**B)**

Suppl. Figure 10: The beta diversity as shown by RDA plot of snails 40 days post exposure to *S. mansoni* (SmBRE, low shedder), *S. mansoni* (SmLE, high shedder), and *S. rodhaini* (Sr). A) Weighted Unifrac distance as shown by RDA plot of the bacterial community across the different parasite exposures. Ordistep model selection for RDA indicated that the additive effect of infection status (F=4.77, p-value =0.001) and parasite exposure (F=8.68, p-value < 0.001) and their interaction effect (F=2.16, p-value =0.027) was significant. B) Unweighted Unifrac distance as shown by RDA plot of the bacterial community across the different parasite exposures. Ordistep model selection for RDA indicated that the additive effect of infection status (F=1.94, p-value =0.027) and parasite exposure (F=9.74, p-value < 0.001) was significant. The triangles indicate centroid values for each parasite exposure, the large grey circle the centroid for all infected samples, and the large grey square the centroid for all uninfected samples. Circles indicate infected samples, squares uninfected samples.


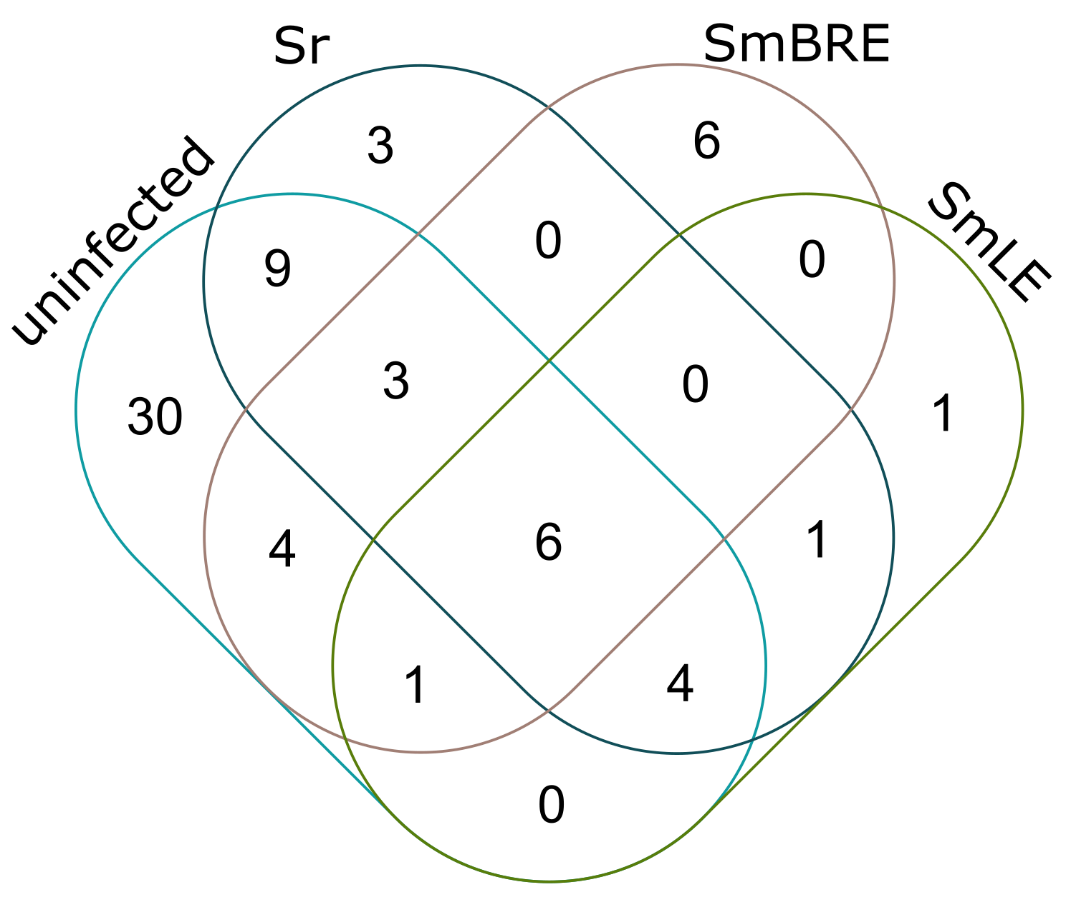


Suppl. Figure 11: Venn diagram showing the shared and unique core ASV members across the samples with different parasite infections (i.e., uninfected N=9, SmBRE N=21, SmLE N=22, and Sr N=18). Core members were determined based on a minimum detection threshold of 0.001 and maximally missing from one sample for that treatment. Venn diagram was generated through https://bioinformatics.psb.ugent.be/webtools/Venn/.

Suppl. Figure 12: Shannon diversity metric for the two co-infection experiments across 2, 10, and 40 days post miracidium exposure (DaysPE). Significant differences as determined through pairwise comparisons between both sub experiments at dayPE 40, and co-exposure with SmLE and SmBRE at 10 daysPE, are indicated with an asterisk.


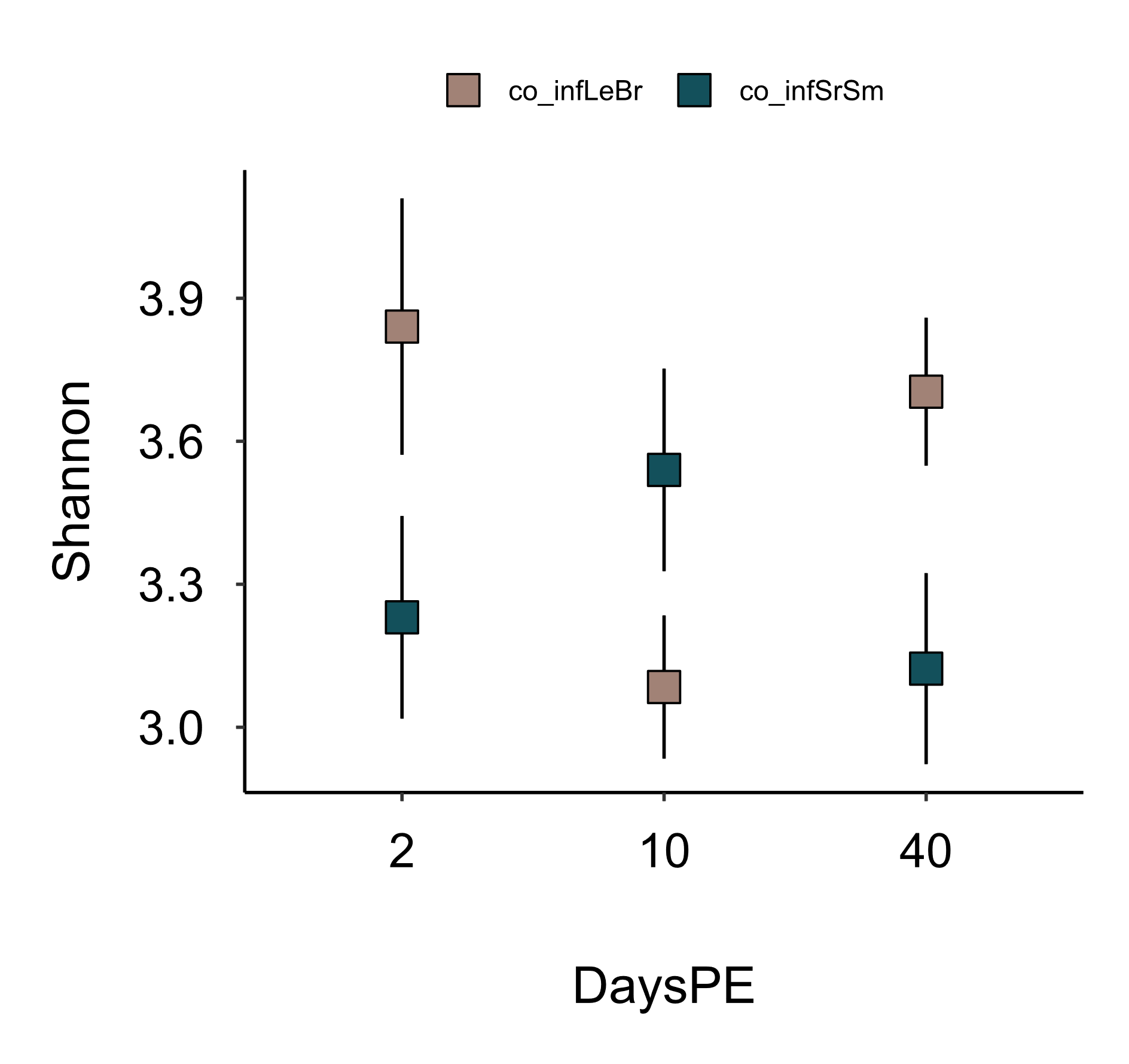


*

*


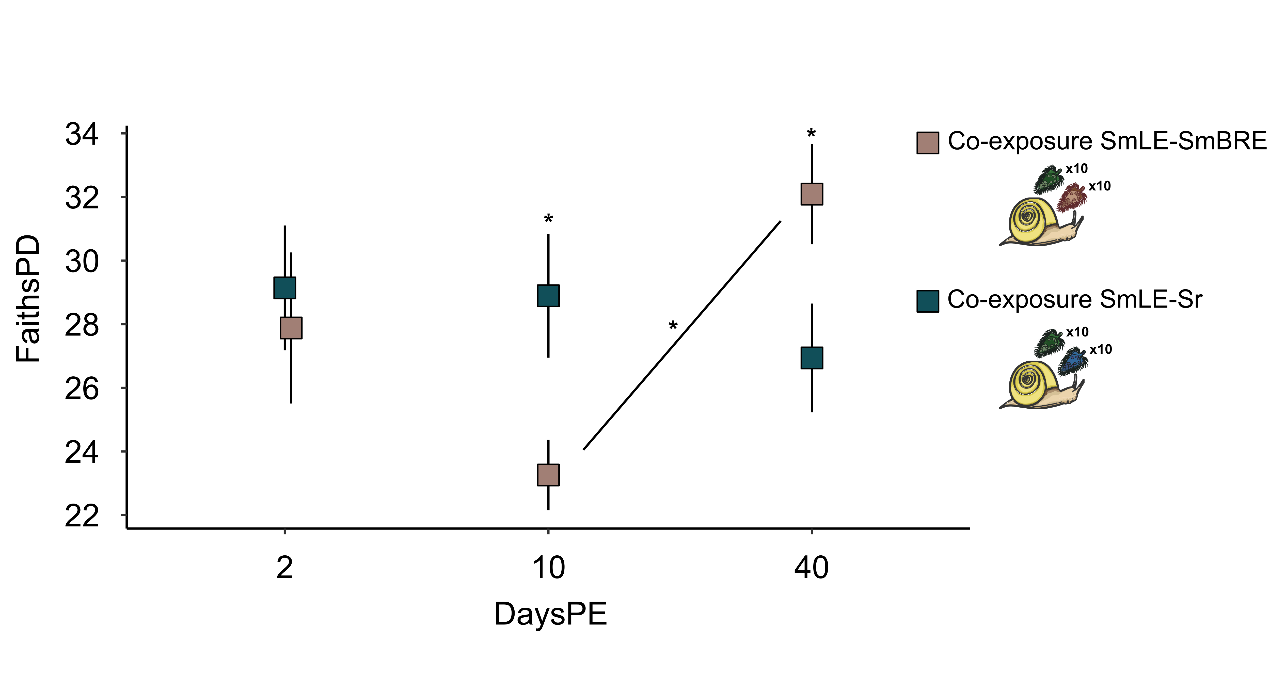

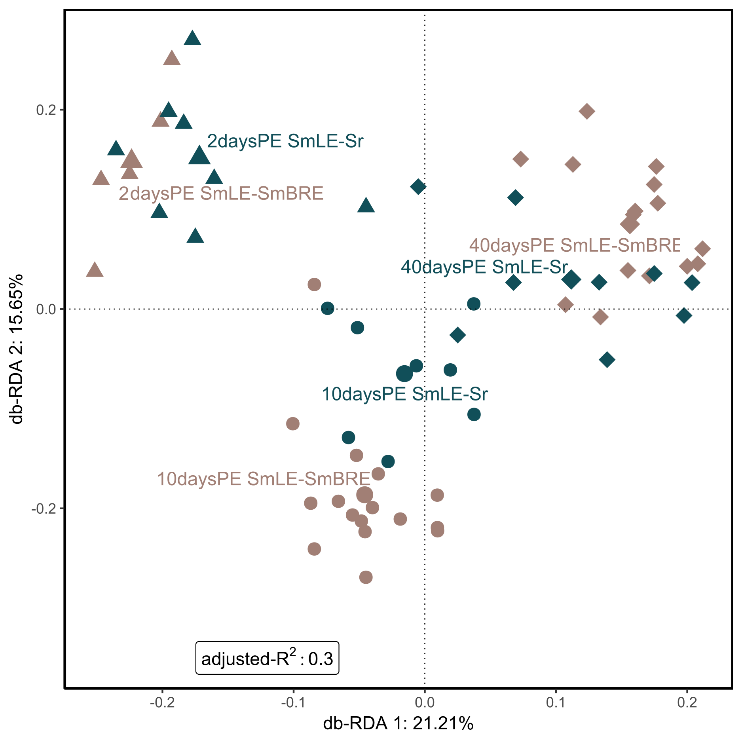

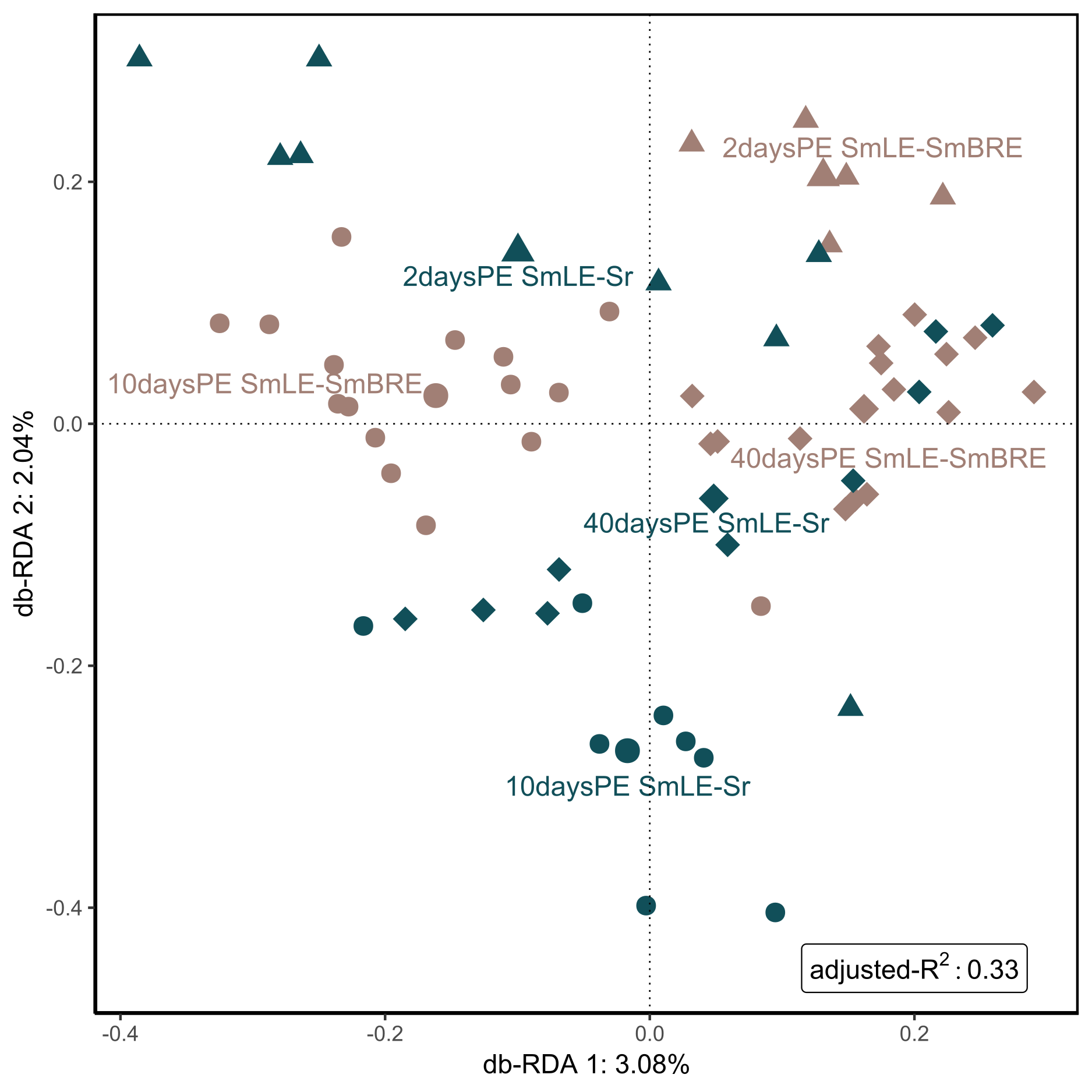


**A)**

**B)**


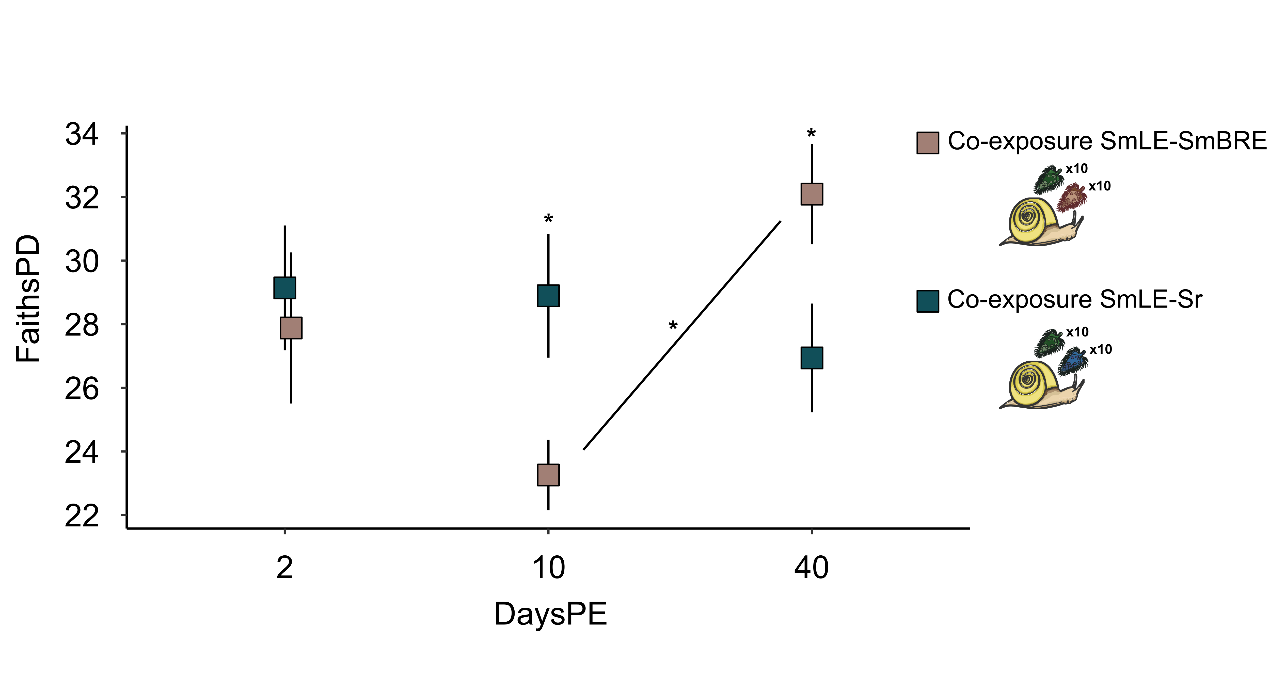

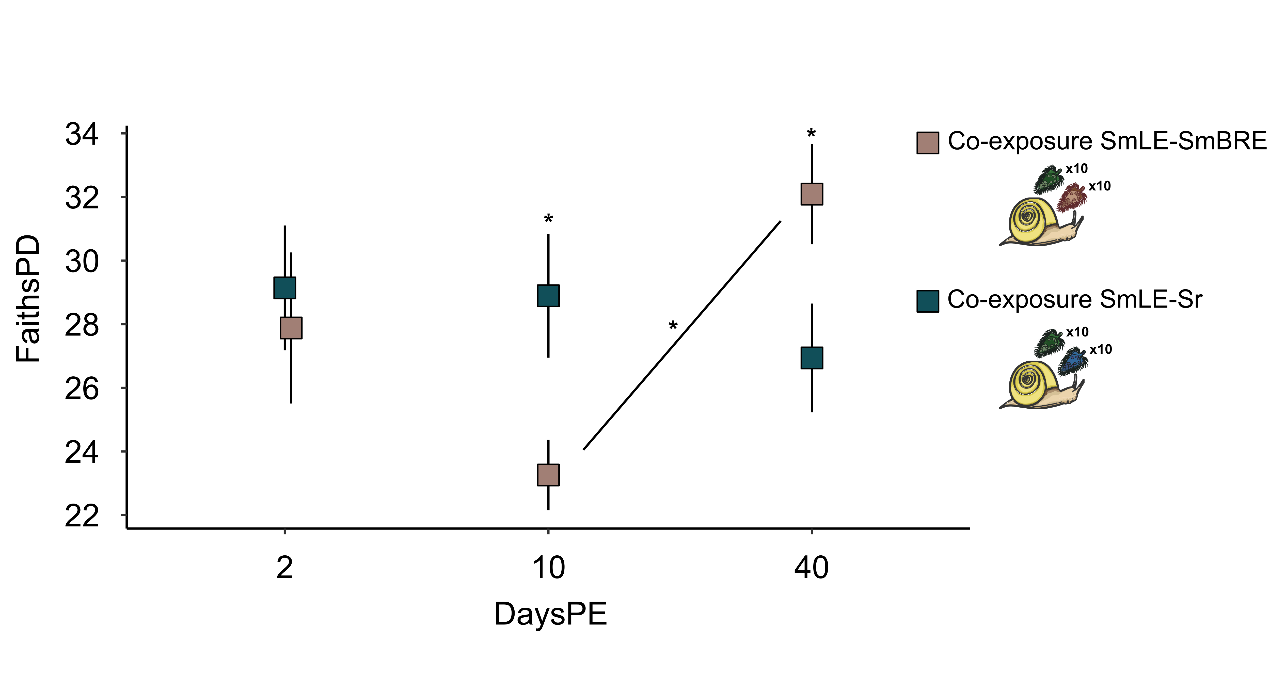


Suppl. Figure 13: The beta diversity as shown by RDA plot during the two co-infection experiments (SmLE & SmBRE, and SmLE & Sr; as indicated by the color code) across 2, 10, and 40 daysPE (irrespective of final infection outcome, as indicated by shape). A) Weighted Unifrac distance as shown by RDA plot of the bacterial community across the different parasite exposures. Ordistep model selection for RDA indicated that daysPE (F=6.67, p-value < 0.001), parasite exposure (F=4.87, p-value < 0.001) and their interaction (F=4.50, p-value < 0.001) significantly affected the bacterial community. B) Unweighted Unifrac distance as shown by RDA plot of the bacterial community across the different parasite exposures. Ordistep model selection for RDA indicated that daysPE (F=9.94, p-value < 0.001), parasite exposure (F=3.28, p-value < 0.001) and their interaction (F=2.80, p-value < 0.001) significantly affected the bacterial community.


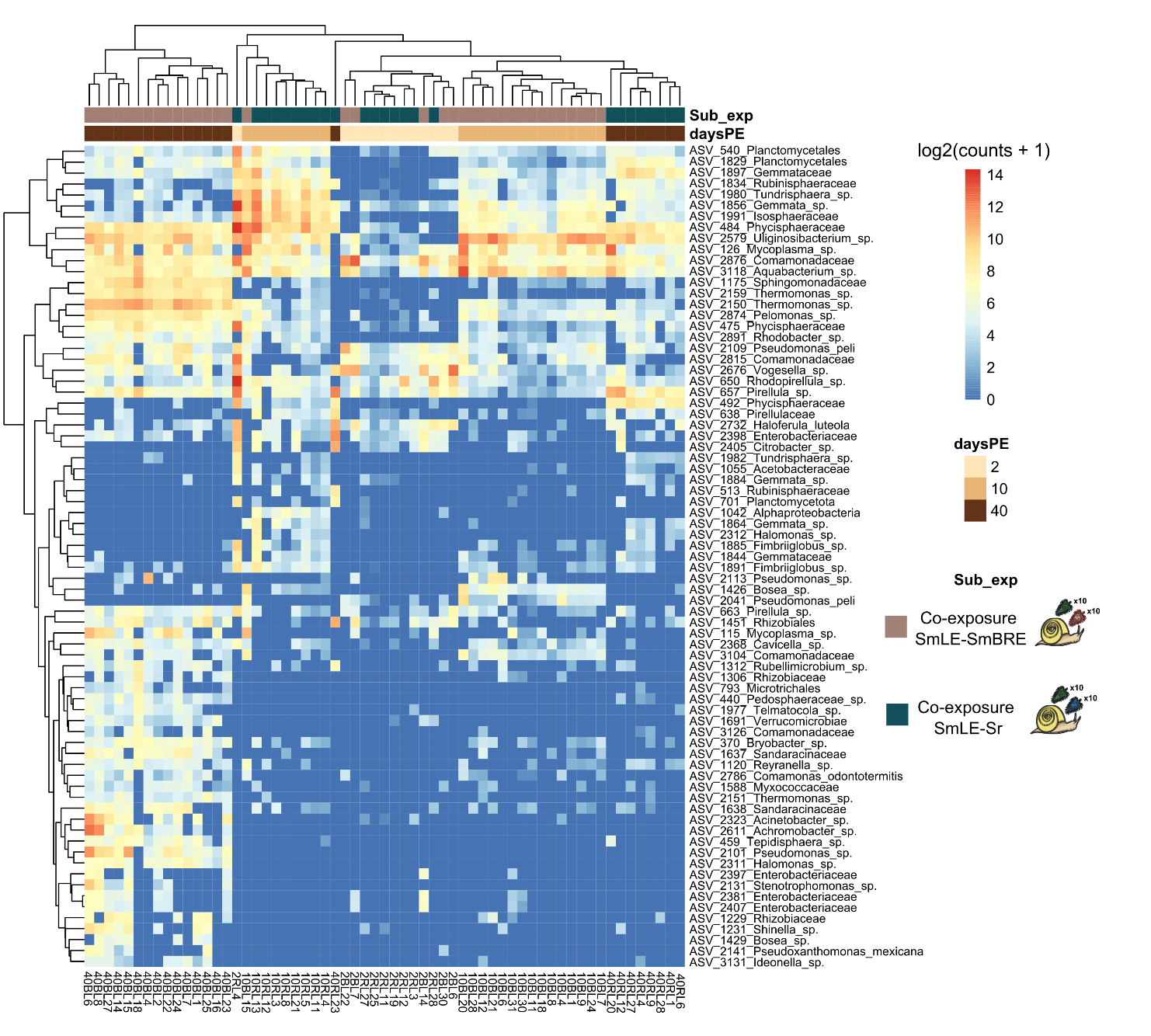
Suppl. Figure 14: Heatmap showing the Log2(read counts+1) for each sample at the ASV level as selected for through DeSeq2 for significantly changing read counts with adjusted p-values <0.05. Sub_exp: co-infection experiment (*S. rodhaini* and *S. mansoni* =co_infSrSm and two populations of *S. mansoni* =co_infLeBr). DaysPE = days post-miracidia exposure (2, 10 and 40). Samples are listed on the X-axis while significantly changing ASVs are listed on the Y-axis. Clustering on top is based on sample similarity based on significantly different ASVs.


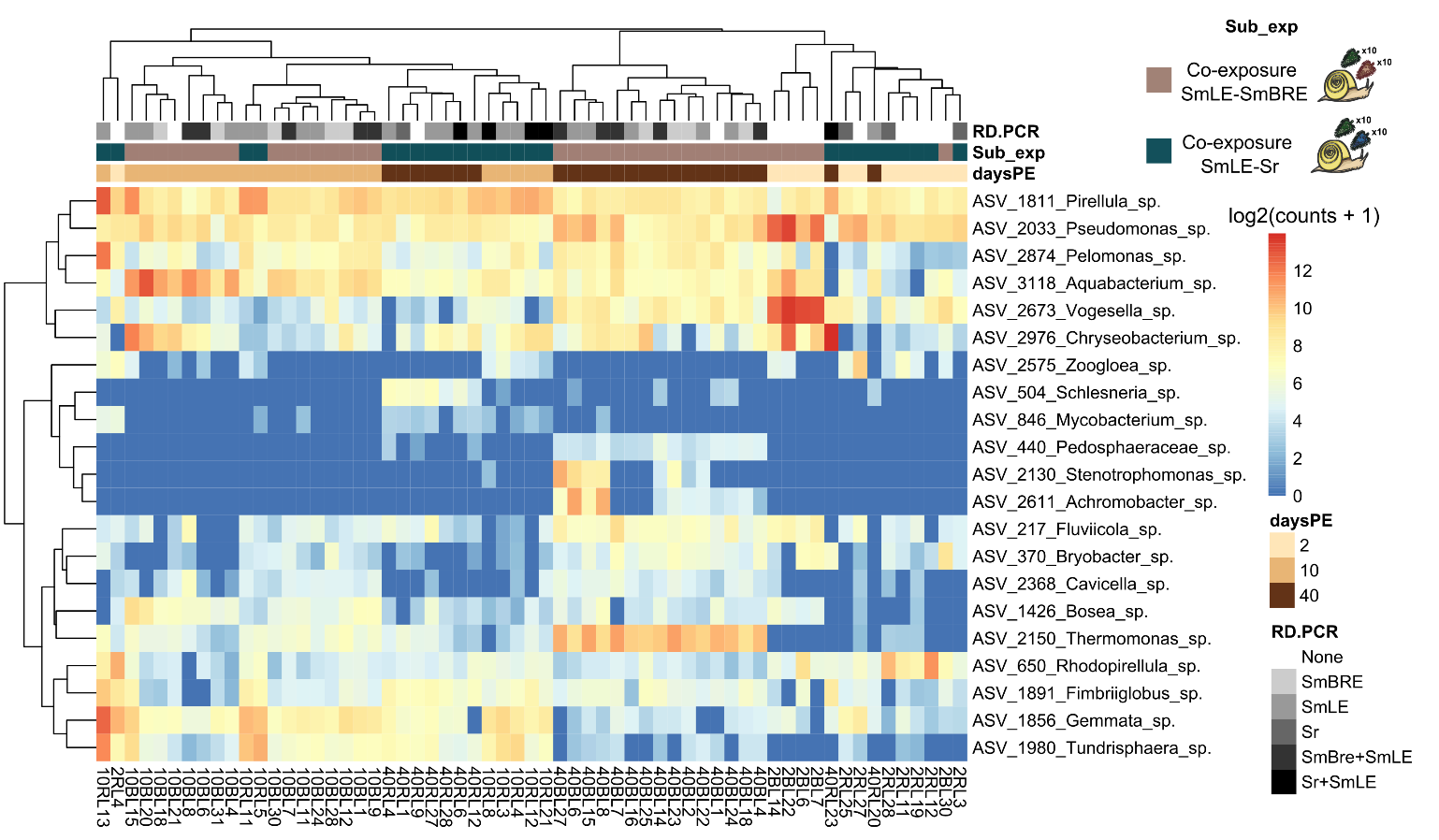


Suppl. Figure 15: Heatmap showing the Log2(read counts+1) for each sample at the genus level as selected for through DeSeq2 for significantly changing read counts with adjusted P-values <0.05. Sub_exp: co-infection experiment (*S. rodhaini* and *S. mansoni* =co_infSrSm and two populations of *S. mansoni* =co_infLeBr). daysPE: Days post miracidia exposure (2, 10 and 40). RD.PCR: lists the molecularly determined (co-)infection status of the sample: *S. mansoni* population BRE (SmBRE), *S. mansoni* population LE (SmLE), and *S. rodhaini* (Sr). Samples are listed in the X-axis while significantly changing genera are listed on the Y-axis.

**References**

1. Schols R, Carolus H, Hammoud C, Mulero S, Mudavanhu A, Huyse T. A rapid diagnostic multiplex PCR approach for xenomonitoring of human and animal schistosomiasis in a “One Health” context. Trans R Soc Trop Med Hyg. 2019 Aug 1;113(11):722–9.

2. Hammoud C, Mulero S, Boissier J, Van Bocxlaer B, Verschuren D, Albrecht C, et al. Simultaneous genotyping of snails and infecting trematode parasites using high-throughput amplicon sequencing. Mol Ecol Resour. 2022;22(2):567–86.

3. Théron A, Gérard C, Moné H. Early enhanced growth of the digestive gland of *Biomphalaria glabrata* infected with *Schistosoma mansoni*: side effect or parasite manipulation? Parasitol Res. 1992;78(5):445–50.
